# Isolating transdiagnostic effects reveals specific genetic profiles in psychiatric disorders

**DOI:** 10.1002/jcv2.70129

**Published:** 2026-05-02

**Authors:** Engin Keser, Wangjingyi Liao, Andrea G. Allegrini, Kaili Rimfeld, Thalia C. Eley, Robert Plomin, Margherita Malanchini

**Affiliations:** ^1^ Social, Genetic and Developmental Psychiatry Centre King's College London London UK; ^2^ School of Biological and Behavioural Sciences Queen Mary University of London London UK; ^3^ Department of Psychology and Language Science University College London London UK; ^4^ Department of Psychology Royal Holloway University of London London UK

**Keywords:** genetic correlations, GWAS, p factor, psychiatric disorders, transdiagnostic

## Abstract

**Background:**

Evidence indicates substantial genetic overlap between psychiatric diagnoses. Accounting for these transdiagnostic effects can sharpen research on disorder‐specific genetic architecture and patterns of comorbidity.

**Methods:**

We applied genomic structural equation modeling to genome‐wide association study summary statistics from 11 major psychiatric disorders to isolate genetic effects shared across disorders (the genomic *p* factor) from residual genetic effects associated with each disorder (*non*‐*p*). Using these *non‐p* summary statistics, we examined SNP heritability, genetic correlations among psychiatric disorders, and genetic correlations with external biobehavioural traits spanning socio‐demographic, anthropometric, health‐related, and psychological domains.

**Results:**

After accounting for transdiagnostic effects, genetic associations between psychiatric disorders changed substantially, with many correlations attenuated and some showing marked shifts in magnitude and direction. Genetic correlations between psychiatric disorders and external biobehavioural traits showed greater specificity.

**Conclusion:**

Removing transdiagnostic effects provides a more nuanced view of the genetic architecture underlying psychiatric disorders and sharpens inference about genetic relationships between disorders and other comorbid traits. This may inform future work on psychiatric classification, prediction, and treatment research.

## Key Points

**What's known?**

- Psychiatric disorders show substantial genetic overlap; complicating efforts to identify disorder‐specific genetic risk.

**What's new?**

- Using genomic structural equation modeling, we isolated transdiagnostic genetic effects that are shared across 11 major psychiatric disorders (*p* factor) to gain further insight into genetic specificity and comorbidity.
- We then examined genetic correlations among psychiatric traits as well as their relationships with other biobehavioural traits.

**What's relevant?**

- Accounting for the transdiagnostic effects substantially altered genetic associations between pairs of disorders, and their genetic correlations with biobehavioural traits.
- These findings may inform future diagnostic frameworks and help guide research into treatment.

## INTRODUCTION

Genetic research has increasingly challenged the current classification of psychiatric disorders as distinct categorical diagnoses by revealing overlaps in their genetic architectures (Plomin, 2022). Notably, the first genome‐wide association study (GWAS) of schizophrenia identified shared genetic loci with bipolar disorder (The International Schizophrenia Consortium, 2009), calling into question their categorical separation in diagnostic manuals such as the DSM‐IV (American Psychiatric Association, 2000). Subsequent large‐scale analyses using linkage disequilibrium score regression (LDSC) (Bulik‐Sullivan, Loh, et al., 2015, Bulik‐Sullivan, Finucane, et al., 2015) revealed significant positive genetic correlations across most major psychiatric diagnoses (Bulik‐Sullivan, Loh, et al., 2015, Bulik‐Sullivan, Finucane, et al., 2015; Cross‐Disorder Group of the Psychiatric Genomics Consortium, 2013; Lee et al., 2019; Selzam et al., 2018), unlike other neurological disorders such as Parkinson's and Alzheimer's disease, which remained genetically distinct (Anttila et al., 2018). More recently, LDSC analysis of 11 major psychiatric diagnoses found that the genetic correlation between schizophrenia and bipolar disorder was 0.68, and the average of 55 genetic correlations between 11 diagnoses was 0.28 (Grotzinger et al., 2022).

The positive genetic manifold between psychiatric diagnoses is consistent with the idea of a general factor of psychopathology (Lahey et al., 2012), called *p* (Caspi & Moffitt, 2018). The *p* factor is commonly conceptualized as describing a general liability to psychopathology (Caspi et al., 2014), and reflects the comorbidities between psychiatric conditions that have been observed concurrently (De Jonge et al., 2018), across the lifespan (Caspi et al., 2020; Plana‐Ripoll et al., 2019), and even across generations (Caspi et al., 2014, 2024). A *p* factor has also emerged from genetic and genomic studies (Allegrini et al., 2020; Grotzinger et al., 2019; Selzam et al., 2018). Shared genetic effects across different disorder dimensions were found to be stable over development, even when considering different measures and reporters (Allegrini et al., 2020). Capturing what cuts across diagnostic categories (i.e., a transdiagnostic approach) has been shown to be more effective in predicting functional and life outcomes than individual diagnoses (Eaton et al., 2023). However, the structure and robustness of a general *p* factor have been found to vary across modeling approaches (Davis et al., 2025; Grotzinger et al., 2022; Watts et al., 2020).

Although the *p* factor provides a useful summary of shared genetic liability across psychiatric disorders, by definition it omits effects that differentiate between diagnoses (Caspi et al., 2014). Correlations between psychiatric disorders are significantly different from unity (Anttila et al., 2018; Grotzinger et al., 2019; Lee et al., 2019), reflecting the presence of both shared and unique components. Therefore, investigating genetic effects after accounting for transdiagnostic liability could provide critical insight into the biology underlying each disorder. Research that separated shared from specific genetic effects revealed novel genetic profiles and biological pathways in neurodevelopmental disorders (Pettersson et al., 2013), alcohol use disorder (Mallard, Karlsson Linnér, et al., 2022, Mallard, Karlsson Linnér, et al., 2022), and substance use disorders in multi‐ancestry analyses (Khan et al., 2025).

In this study, we applied genomic structural equation modeling (genomic SEM) (Grotzinger et al., 2019) to isolate transdiagnostic genetic effects across 11 psychiatric disorders (*p*) from genetic effects associated with individual conditions. As shorthand, we refer to these residual genetic effects as *non‐p* to describe that they capture genetic variance beyond *p*. We used summary statistics from these *non‐p* GWASs to test the hypothesis that partialling out transdiagnostic effects will provide us with novel insight into the genetic architecture of psychiatric disorders and their co‐occurrences. Therefore, we investigated changes in the heritability of each condition and genetic correlations between psychiatric disorders after removing transdiagnostic effects. We also examined changes in the genetic correlations between *non‐p* and other biobehavioural traits, spanning socio‐demographic, anthropometric, health, and psychological domains. Lastly, we conducted gene‐based and tissue enrichment analyses to examine how accounting for transdiagnostic liability alters the biological pathways associated with psychiatric risk.

## METHODS

The article is accompanied by Supporting Information, and the study followed a preregistered analysis plan. Deviations from the registered protocol are described in Supporting Information S1: Appendix S1.

### GWAS summary statistics

We used the most recent publicly available GWAS summary statistics for 11 major psychiatric disorders (details in Supporting Information S2: Table S1). All GWASs were based on samples of European ancestry.

### Genomic SEM

**FIGURE 1:**
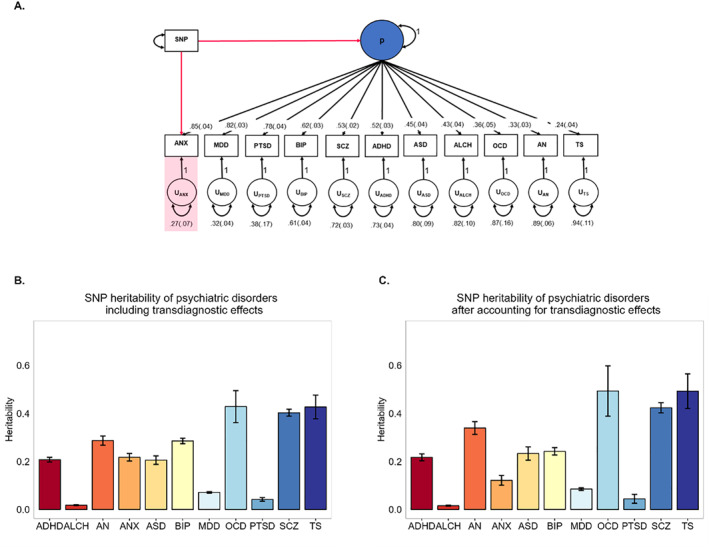
Isolating transdiagnostic genetic effects from 11 major psychiatric disorders. (A) Standardized results for a common factor model of genomic p. Each square indicates observed variables (i.e., the summary statistics for each of the 11 major psychiatric disorders) and circles represent latent variables that are statistically inferred from the data (i.e., genomic p‐factor). One‐headed arrows are standardized factor loadings, representing regression relations with the arrow pointing from the predictor variable to the outcome variable. Covariance relationships between variables are represented as two‐headed arrows linking the variables. Residual variances of a variable are represented as a two‐headed arrow connecting the variable to itself. SEs are shown in parentheses. The red arrows linking the SNP to both the p‐factor and PTSD provide an example of the model used to partition genetic variance associated with transdiagnostic effects from the genetic variance specific to each disorder. We ran the model 11 times, isolating transdiagnostic effects from each psychiatric condition at a time. (B, C) SNP‐based heritability estimates before (Panel B, on the liability scale) and after (Panel C, on the observed scale) accounting for transdiagnostic effects. Error bars indicate standard errors. ADHD = attention‐deficit hyperactivity disorder; ALCH = problematic alcohol use; AN = anorexia nervosa; ANX = anxiety disorder; ASD = autism spectrum disorder; BIP = bipolar disorder; MDD = major depressive disorder; OCD = obsessive‐compulsive disorder; PTSD = post‐traumatic stress disorder; SCZ = schizophrenia; TS = Tourette syndrome; *p* = general psychopathology factor.

We used genomic SEM to conduct multivariate GWAS analysis of 11 major psychiatric disorders (Figure 1). This approach allowed us to model transdiagnostic genetic effects shared across all disorders and to capture residual genetic variance associated with each disorder after accounting for *p*. Genomic SEM (Grotzinger et al., 2019) is a statistical framework that applies structural equation modeling to GWAS summary statistics to model patterns of associations between complex traits and is robust to sample overlap and imbalanced sample sizes across studies (Grotzinger et al., 2019).

Genomic SEM uses multivariable LD score regression (LDSC) to estimate the genetic covariance matrix and sampling covariance matrix. We applied quality control filters for this step using the defaults in Genomic SEM, including restricting SNPs to those present in HapMap3 with a minor allele frequency >1% and information score >0.9. The LD weights used for LDSC were calculated using the European subsample of the 1000 Genomes Phase3 (The 1000 Genomes Project Consortium et al., 2015); excluding the major histocompatibility complex (MHC) due to complex LD structures in this region that can bias estimates. When calculating heritability estimates on the liability scale for the uncorrected psychiatric disorders, we used the sum of effective sample sizes, and a sample prevalence of 0.5 to reflect that the corrected sample size already accounts for sample ascertainment. Following these quality control steps, 3,746,806 SNPs were retained across all 11 disorders. Further details on processing of summary statistics in Genomic SEM and calculating effective sample size are provided in Supporting Information S1: Appendix S2.

To assess the extent to which our results were robust to differences in model implementation, we compared the single‐step genomic SEM approach described above, which estimates *p* and *non‐p* simultaneously, with a two‐step approach. In this alternative approach to partition variance, a genomic *p* factor was estimated first, the *p* summary statistics were then exported and used in a second model to remove transdiagnostic effects from each disorder using GWAS‐by‐subtraction (Demange et al., 2021) (See Supporting Information S1: Appendix S3 and Figure S1).

### Independent SNPs, genes, and enrichment and pathway analysis (MAGMA)

To identify independent hits from the 11 *non‐p* GWASs, we applied a pruning approach using a window of 250 kb and a linkage disequilibrium (LD) threshold of *r* ^2^ < 0.1, implemented using the clumping functionality in PLINK v.1.9 (Purcell et al., 2007) with LD statistics obtained from the 1000 Genomes Phase 3 reference panel. Independent significant SNPs were considered novel if the same rsID had not been previously reported as genome‐wide significant in the corresponding original disorder GWAS. Novelty was thus defined at the SNP level relative to the corresponding GWAS, rather than at the locus level.

We used MAGMA within the FUMA framework (v1.5.6) (Watanabe et al., 2017) to map SNPs to genes based on their position. To allow the inclusion of nearby regulatory variants, we considered all SNPs within a 35 kb upstream and 10 kb downstream window of the gene transcription region (The Network and Pathway Analysis Subgroup of the Psychiatric Genomics Consortium, 2015). We performed genome‐wide gene‐based association tests using MAGMA. The gene‐based test combines results from multiple SNPs within a gene to assess the association between the gene and the disorder, while accounting for LD between SNPs. LD information was obtained from the 1000 Genomes Phase 3 EUR reference panel, and Bonferroni correction was applied to identify genes with genome‐wide significance.

We used MAGMA to conduct tissue‐specific gene‐set analysis and gene property analysis. Gene‐set analyses assessed whether genes within an annotated set exhibited stronger associations with the disorder compared to other genes. Meanwhile, the tissue specificity test examined the relationship between tissue‐specific gene expression profiles and disorder‐gene associations. The gene‐set analyses were performed using curated gene sets and Gene Ontology (GO) terms obtained from the Molecular Signatures Database v2023.1Hs. For the MAGMA gene property analysis, tissue expression profiles were obtained from GTEx v8 (comprising 53 tissue types) and BrainSpan (brain samples at 11 general developmental stages), available in FUMA. Gene sets and tissue types were considered significant at a Bonferroni‐corrected *p*‐value threshold of <0.05.

### SNP heritabilities and genetic correlations

SNP‐based heritabilities and pairwise genetic correlations among the 11 disorders were estimated using LDSC (Bulik‐Sullivan, Loh, et al., 2015, Bulik‐Sullivan, Finucane, et al., 2015) implemented in genomic SEM. Analyses were conducted both before and after residualizing disorder summary statistics on the genomic *p*. We also estimated genetic correlations between each disorder –both uncorrected and *p*‐corrected– and 34 external traits spanning socio‐demographic, anthropometric, health‐related, and psychological domains commonly examined in psychiatric genetic studies. These traits were selected based on previous studies that investigated cross‐trait genetic associations (Demange et al., 2021; Grotzinger et al., 2022), as well as the public availability of well‐powered GWAS summary statistics.

The LD scores used were computed using 1,215,002 SNPs present in the HapMap3 reference panel, excluding the MHC region on chromosome 6. The differences between genetic correlations with external traits before and after partialling out genetic effects associated with the *p* factor were assessed using a two‐stage method (Coleman et al., 2020). We used this approach to, first, assess differences in genetic correlations using a two‐sample *z*‐test, and then we compared significant differences (*p* < 0.05) using a block‐jackknife correction (see Coleman et al., 2020 for a detailed description). The jackknife estimates were then corrected for multiple testing using Benjamini‐Hochberg False Discovery Rate (FDR).

## RESULTS

### Isolating transdiagnostic genetic signal from 11 major psychiatric disorders

We used genomic SEM to construct a genomic *p* factor using the most recent publicly available summary statistics from GWAS of 11 major psychiatric disorders (see Supporting Information S2: Table S1). Genetic correlations between the 11 disorders are presented in Supporting Information S2: Table S2. We found a positive manifold of genetic correlation (*r*_g_) among most disorders (mean *r*_g_ of 0.29). Estimates ranged between −0.11 for the *r*_g_ between obsessive compulsive disorder (OCD) and attention deficit hyperactivity disorder (ADHD) and 0.90 between anxiety and major depressive disorder (MDD).

To capture transdiagnostic genetic effects across these 11 disorders, we fitted a common factor model to the genetic covariance matrix. In this model, all disorders loaded on a single common factor (i.e., the *p* factor; top half of Figure 1A; CFI = 0.82; SRMR = 0.12) (See Supporting Information S1: Appendix S4). The model additionally captured residual genetic variance associated with each disorder after accounting for transdiagnostic effects (i.e., *non‐p*; bottom half of Figure 1A). We then conducted GWAS on *p* and on the residual variance in each psychiatric disorder. Figure 1A illustrates the model used to capture genetic variance in post‐traumatic stress disorder (PTSD) after accounting for transdiagnostic genetic effects; this procedure was repeated 11 times to capture genetic variance in each psychiatric disorder.

After accounting for transdiagnostic genetic effects, we identified independent significant hits for seven out of eleven psychiatric disorders. The largest number of independent hits was observed for the *non‐p* GWAS of schizophrenia (118 independent SNPs, 27 of which were novel SNP associations that had not emerged as significant in the original GWAS), followed by BIP (22, 13 novel SNP associations), MDD (14, 11 novel SNP associations), ADHD (12, 8 novel SNP associations), anorexia nervosa (AN) (10, 8 novel SNP associations), ALCH (3, 1 novel SNP associations) and autism spectrum disorder (ASD) (2, no novel SNP associations). Some of these novel SNP associations had been uncovered by GWAS of psychiatric disorders other than the 11 included in our model, or by GWAS of non‐psychiatric traits, while others had not been previously reported. For example, focusing on schizophrenia, 7 of the 27 novel SNP associations had been identified by previous genomic studies of schizophrenia (Bhattacharya et al., 2023; Chen et al., 2019; Goes et al., 2015; Ikeda et al., 2019), 6 had been previously reported as SNPs associated with physiological or psychological traits (i.e.,; body mass index (BMI) (Christakoudi et al., 2022; Sakaue et al., 2021) and intelligence (Hill et al., 2019)), and 14 SNP associations had not been previously reported. Details regarding the novel SNPs identified in the other six *non‐p* GWASs are presented in Supporting Information S2: Tables S3–S9. Manhattan plots for the 11 non‐*p* GWASs are shown in Supporting Information S1: Figures S2–S12.

We used MAGMA (De Leeuw et al., 2015) in FUMA to conduct gene‐level and gene‐set analyses and identify biological pathways linked to the genes associated with each major psychiatric disorder before and after accounting for *p*, as well as to analyze tissue type enrichment. All results reported are Bonferroni‐corrected to reduce multiple comparison problems. We found that, after accounting for transdiagnostic genetic effects, 316 genes were associated with SCZ, 63 with BIP, 44 with MDD, 29 with ADHD, 37 with AN, 5 with ASD, and 1 with ALCH. Only SCZ, BIP and MDD showed significant enriched gene sets after accounting for *p*. SCZ *non‐p* showed the greatest enrichment: it was enriched in six gene‐sets related to neuron system (Supporting Information S2: Tables S10, S11). The full results are reported in Supporting Information S2: Tables S10‐S19.

We tested whether common variants in genes specifically expressed in 53 Genotype‐Tissue Expression (GTEx) tissues were enriched in their effects on psychiatric disorders (SCZ, BIP, MDD, ADHD, ALCH, ASD, AN) after accounting for transdiagnostic effects. Genes predominantly expressed in the brain cortex and other brain‐specific tissues were enriched in MDD, BIP, SCZ, ADHD, ASD and AN (Supporting Information S1: Figures S13–S26, and Supporting Information S2: Tables S20–S47). Enrichment patterns were overall consistent between psychiatric disorders before and after removing transdiagnostic signals, with a few exceptions. First, brain development stages enrichment results showed that, for SCZ, the early‐to‐late prenatal stages were enriched before accounting for *p* but no longer enriched after removing transdiagnostic effects. Late infancy remained the most enriched developmental stage for SCZ *non‐p*, showing the strongest associations (Supporting Information S1: Figure S18, and Supporting Information S2: Tables S21, S23). Second, for ASD *non‐p* tissue property enrichment analyses showed that 10 brain regions were enriched, with the substantia nigra showing the strongest signal if compared to four brain regions before accounting for transdiagnostic genetic effects (Supporting Information S1: Figure S22, and Supporting Information S2: Tables S44, S46).

### SNP heritability of major psychiatric disorders independent of p

We used LDSC to estimate SNP‐based heritability (h^2^) for the 11 disorders before and after partialling out the genetic variance associated with *p*. Figure 1B shows the SNP‐based h^2^ estimates for the 11 disorders on the liability scale and Figure 1C shows the residual variance in each psychiatric disorder after accounting for genomic *p*, on the observed scale (see also Supporting Information S2: Table S48). Although it is challenging to compare heritability estimates measured on different scales, the pattern of results is mostly preserved across all disorders. For example, both before and after isolating the variance associated with genomic *p*, the highest SNP h^2^ were observed for OCD and Tourette syndrome (TS) and the lowest estimates for ALCH and PTSD.

### Removing transdiagnostic genetic effects significantly changed genetic relationships between psychiatric disorders

**FIGURE 2:**
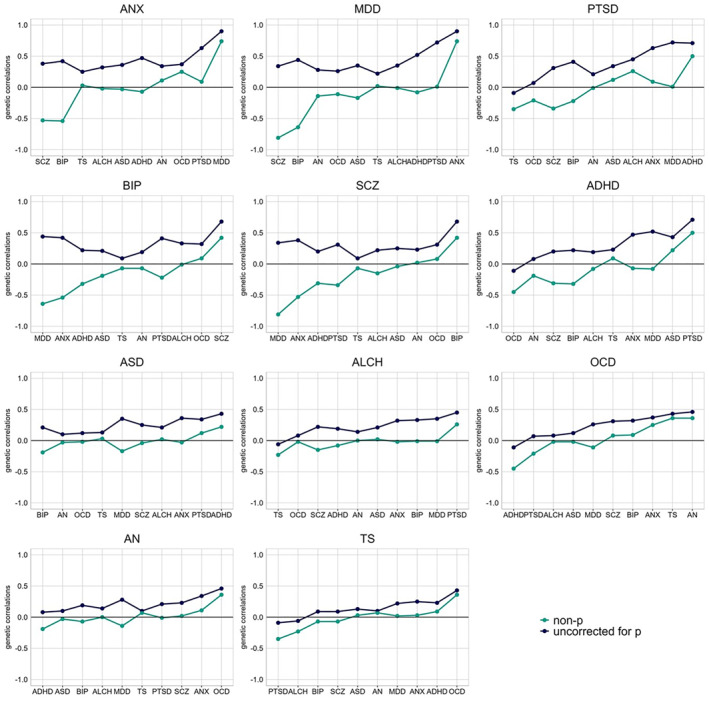
Changes in the landscape of genetic correlations between psychiatric disorders after accounting for transdiagnostic effects. For every psychiatric disorder, we present genetic correlations with the other 10 psychiatric conditions uncorrected for p (blue line) and the genetic correlations with the other psychiatric disorders after removing the genetic variance captured by the p factor (*non‐p*; green line). Disorders are presented ordered by their loading on the p factor, starting from the highest‐loading disorders in the top row (i.e., PTSD, MDD and ANX) to those that shared the least amount of genetic variance with the p factor in the bottom row (i.e., AN and OCD). Correlations were estimated using LDSC within Genomic SEM. ADHD, attention‐deficit hyperactivity disorder; ALCH, problematic alcohol use; AN, anorexia nervosa; ANX, anxiety disorder; ASD, autism spectrum disorder; BIP, bipolar disorder; MDD, major depressive disorder; OCD, obsessive‐compulsive disorder; PTSD, post‐traumatic stress disorder; SCZ, schizophrenia; TS, Tourette syndrome.

Figure 2 compares pairwise genetic correlations across the 11 psychiatric disorders before and after removing the genetic variance in each disorder that is accounted for by *p* (see Supporting Information S2: Table S49). Overall, genetic correlations dropped substantially after accounting for *p*: the mean genetic correlation across all pairwise associations decreased from 0.29 to −0.05.

However, the observed reductions were not uniform across disorder pairs, as visualized in Figure 2 where the blue and green lines are not parallel. For example, consider the genetic correlations between ADHD with PTSD and MDD. Uncorrected for p –as indicated by the blue line in Figure 2– the genetic correlation between ADHD and PTSD is 0.71 (95% CIs = [0.85, 0.58]). After accounting for genomic p –as shown by the green line in figure 2– the genetic correlation between ADHD and PTSD declines to 0.50 (95% CIs = [0.73, 0.28]). This suggests that the substantial genetic correlation between ADHD and PTSD is only partly due to transdiagnostic genetic risk. In contrast, when considering the relationship between ADHD and MDD, their genetic correlation drops from 0.52 (95% CIs = [0.58, 0.46]) to −0.08 (95% CIs = [0.02, −0.18]). This suggests that the genetic relationship between ADHD and MDD is nearly entirely mediated by genetic risk that is not specific to either condition.

Three key patterns of change were observed after accounting for transdiagnostic genetic effects. First, for many disorder pairs, genetic correlations were reduced, although reductions varied substantially in magnitude. For some disorders, reduced correlations remained moderate to strong, such as between ADHD and PTSD (*r*_g_ = 0.50, 95% CIs = [0.73, 0.28]), ANX and MDD (*r*_g_ = 0.74, 95% CI [0.89, 0.59]) and between SCZ and BIP (*r*_g_ = 0.42, 95% CI [0.50, 0.35]), indicating shared genetic liability beyond that captured by the genomic *p* factor. For other disorders, correlations were markedly attenuated, including the association between ADHD and MDD (attenuated from *r*_g_ = 0.52, 95% CI [0.58, 0.46] to *r*_g_ = −0.08 95% CI [0.02, −0.18]), PTSD and ANX (from *r*_g_ = 0.63, 95% CI [0.79, 0.47] to *r*_g_ = 0.09, 95% CI [0.43, −0.25]), and ASD and ANX (from *r*_g_ = 0.36, 95% CI [0.47, 0.25] to *r*_g_ = −0.03, 95% CI [0.16, −0.22]).

Second, for disorder pairs that shared weak negative associations or correlations that were not significant before accounting for genomic *p*, associations became significantly stronger in the negative direction after removing transdiagnostic effects. For example, the genetic correlation between OCD and ADHD increased in magnitude from *r*_g_ = −0.11 (95% CI [0.01, −0.23]) to *r*_g_ = −0.45 (95% CI [−0.28, −0.62]), and between TS and PTSD from −0.09 (95% CI [0.10, −0.29]) to −0.35 (95% CI [0.01, −0.72]).

Third, associations between some disorder pairs changed in both magnitude and direction after accounting for genomic *p*. Most notably, the genetic correlation between MDD and SCZ shifted from a positive and moderate association, *r*_g_ = 0.34 (95% CI [0.39, 0.28]) to a strong negative association. *r*_g_ = −0.81 (95% CI [−0.72, −0.90]). A similar change was observed for the association between MDD and BIP from *r*_g_ = 0.44 (95% CI [0.50, 0.38]) to *r*_g_ = −0.64 (95% CI [‐0.53, −0.75]). Such reversals reflect changes in conditional genetic associations after accounting for transdiagnostic effects and do not necessarily imply antagonistic etiological relationships.

Results were robust to model implementation, with consistent patterns of genetic correlations observed when we modeled *non‐p* genetic variance using the two‐step GWAS‐by‐subtraction approach (See Methods and Supporting Information S1: Appendix S3 and Figure S27).

### Genetic associations with other traits

In addition to removing transdiagnostic genetic effects from the relationships between psychiatric disorders, we also compared genetic correlations between each of the 11 psychiatric disorders—uncorrected and corrected for *p*—and 34 traits that are not psychiatric disorders. These 34 traits spanned socio‐demographic, anthropometric, health‐related, and psychological domains.

**FIGURE 3:**
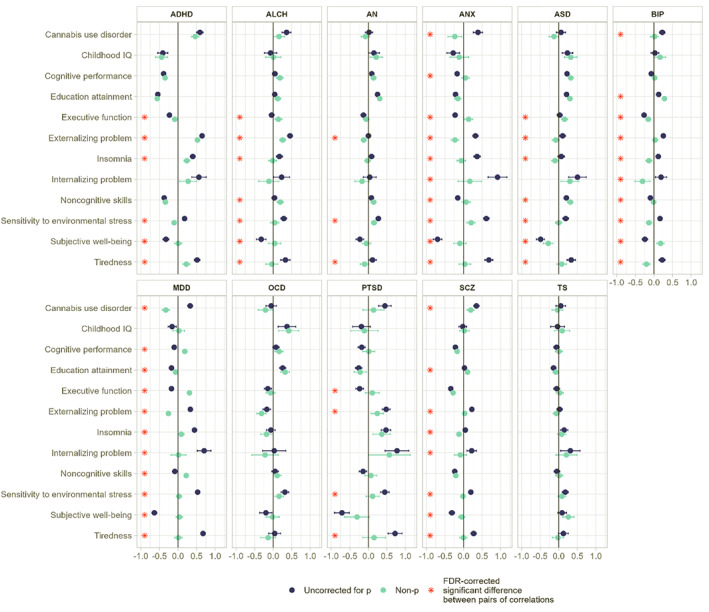
Genetic correlations between 11 major psychiatric disorders and psychological traits. The dots represent genetic correlations estimated using LDSC regression. Correlations between psychiatric disorders uncorrected for *p* are in blue, while correlations between psychiatric disorders corrected for *p* (*non‐p*) in green. Error bars represent 95% confidence intervals. Red asterisks indicate a statistically significant (FDR‐corrected *p* < 0.05, two‐tailed test) differences in the magnitude of the correlation with disorders uncorrected for *p* versus disorders corrected for p. Exact *p* values for all associations are reported in Supporting Information S2: Table S50. The false discovery rate correction was applied based on all genetic correlations tested (including those reported in Supporting Information S1: Figures S28, S29). Source GWASs are listed in Supporting Information S2: Table S51.

Figure 3 presents the changes in genetic correlations with psychological traits. Associations were generally attenuated after accounting for *p*, with the magnitude of change varying across disorders. For example, genetic correlations between sensitivity to environmental stress and several psychiatric disorders were substantial prior to correction but markedly reduced or negligible after accounting for *p* (e.g., MDD (attenuated from *r*_g_ = 0.53, 95% CI [0.49, 0.57] to *r*_g_ = 0.03, 95% CI [−0.05, 0.10]); SCZ (from *r*_g_ = 0.20, 95% CI [0.15, 0.24] to *r*_g_ = −0.02, 95% CI [−0.08, 0.05]; ASD (from *r*_g_ = 0.19, 95% CI [0.11, 0.26] to *r*_g_ = −0.002, 95% CI [−0.09, 0.08]; ALCH (from *r*_g_ = 0.29, 95% CI [0.21, 0.36] to *r*_g_ = 0.04, 95% CI [−0.07, 0.16])). Similar attenuations were observed for other traits, including subjective wellbeing, loneliness, tiredness, and insomnia.

**FIGURE 4:**
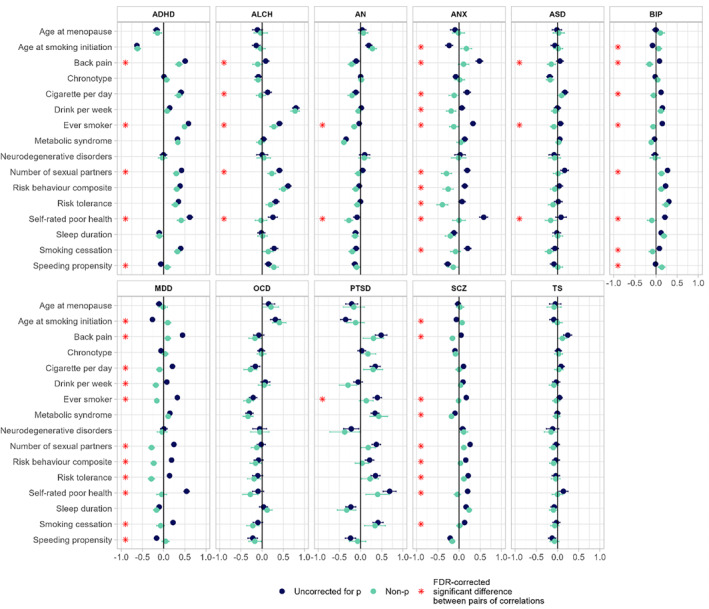
Genetic correlations between 11 major psychiatric disorders and health‐related traits. The dots represent genetic correlations estimated using LDSC regression. Correlations with psychiatric disorders uncorrected for p (“original”) are in blue, with psychiatric disorders corrected for p (*non‐p*) in green. Error bars represent 95% confidence intervals. Red asterisks indicate a statistically significant (FDR‐corrected *p* < 0.05, two‐tailed test) differences in the magnitude of the correlation with disorders uncorrected for p versus disorders corrected for p. Exact *p* values for all associations are reported in Supporting Information S2: Table S50. The false discovery rate correction was applied based on all genetic correlations tested (including those reported in Supporting Information S1: Figures S28, S29).

Figure 4 shows changes in the genetic correlations with health‐related traits, where genetic correlations corrected for p were generally lower than uncorrected estimates. Notably, correlations with self‐reported poor health, number of sexual partners, and cigarettes per day were significantly reduced for multiple disorders (see Supporting Information S2: Table S50). The results for socio‐demographic and anthropometric traits are shown in Supporting Information S1: Figures S28 and S29. All correlations are detailed in Supporting Information S2: Table S50.

Although attenuation was the predominant pattern, some associations strengthened or reversed after accounting for *p*. For example, the genetic correlation between BIP and SCZ and educational attainment increased after correction (BIP increased from *r*_g_ = 0.13, 95% CI [0.09, 0.16] to *r*_g_ = 0.28, 95% CI [0.23, 0.33]; SCZ from *r*_g_ = 0.02, 95% CI [−0.01, 0.06] to *r*_g_ = 0.11, 95% CI [0.06, 0.15]). In contrast, several associations reversed in direction, including correlations between MDD and multiple cognitive and risk‐related traits (cognitive performance: *r*_g_ = −0.10, 95% CI [−0.14, −0.06] to *r*_g_ = 0.18, 95% CI [0.12, 0.24]; executive functions: *r*_g_ = −0.18, 95% CI [−0.22, −0.13] to *r*_g_ = 0.31, 95% CI [0.25, 0.37]; noncognitive skills: *r*_g_ = −0.09, 95% CI [−0.13, −0.05] to *r*_g_ = 0.22, 95% CI [0.16, 0.28]; risk tolerance: *r*_g_ = 0.14, 95% CI [0.08, 0.20] to *r*_g_ = −0.29, 95% CI [−0.37, −0.21]), as well as between ANX and risk‐taking behaviors (risk behavior composite: *r*_g_ = 0.14, 95% CI [0.07, 0.20] to *r*_g_ = −0.26, 95% CI [−0.38, −0.13]; risk tolerance: *r*_g_ = 0.07, 95% CI [−0.01, 0.15] to *r*_g_ = −0.39, 95% CI [−0.52, −0.26]) (see Supporting Information S2: Table S50).

## DISCUSSION

We isolated transdiagnostic genetic effects across 11 major psychiatric disorders from residual genetic effects associated with each psychiatric condition. Consistent with our preregistered predictions, accounting for transdiagnostic genetic effects markedly altered genetic correlations both among psychiatric disorders and with external biobehavioural traits, particularly for psychological and health‐related traits, and revealed heterogeneity in residual SNP‐based heritability across disorders. Beyond the preregistered analyses, exploratory investigations identified differences in the genetic architecture of each disorder, including novel genome‐wide significant SNP associations for four psychiatric disorders—schizophrenia, bipolar disorder, ADHD and anorexia nervosa—as well as changes in tissue enrichment profiles. By isolating transdiagnostic genetic effects and considering residual genetic risk, we provide novel insight into the genetic architecture, biology and comorbidity between psychiatric conditions that can inform diagnostic nomenclature and treatment research.

Despite reduced statistical power relative to uncorrected GWASs, accounting for transdiagnostic effects allowed us to identify several novel loci associated with psychiatric risk. For example, among the 27 novel SNP associations identified with SCZ, one top signal (rs693906) has not been previously reported by GWASs before, but was an intronic SNP in a gene (SLC44A4) associated with SCZ in a Japanese sample (Yamada et al., 2015). Such novel SNP associations may provide leads for future investigations into biological pathways more specific to SCZ. Gene‐ and tissue‐level analyses further revealed changes in enrichment patterns after accounting for transdiagnostic effects, primarily for SCZ and BIP, indicating that shared genetic liability contributes substantially to previously observed biological signals. SNP heritabilities for most disorders were largely preserved after conditioning on *p*, with notable reductions observed for MDD and ANX, consistent with these conditions having a larger proportion of their heritable variance captured by transdiagnostic genetic effects.

The most striking findings concerned changes in genetic correlations between psychiatric disorders. Accounting for *p* substantially altered both the magnitude and, in some cases, the direction of genetic relationships. For some disorder pairs, such as ANX and PTSD, previously strong positive genetic correlations were markedly attenuated and in some cases were no longer significant. This suggests that their overlap could largely be attributed to transdiagnostic genetic liability rather than a relationship specific to each pair of conditions. In contrast, other disorder pairs, for example, SCZ and BIP, or ANX and MDD, retained substantial correlations, pointing to additional genetic risk common to these conditions beyond transdiagnostic risk. Importantly, these differences were not explained solely by the extent to which variance in each disorder was captured by the genomic *p* factor, reflected in differences in factor loadings. This is exemplified by the markedly different pattern of changes in genetic relationships observed for PTSD and MDD, two disorders with comparable high loadings on the genomic *p* factor. This highlights heterogeneity in the genetic architecture of each disorder once transdiagnostic effects are statistically accounted for.

In some cases, genetic correlations shifted from strongly positive to strongly negative after accounting for *p*, most notably the association between SCZ and MDD, and MDD and BIP. These reversals indicate that much of the apparent overlap in uncorrected analyses reflects shared transdiagnostic liability, while residual genetic components can exhibit qualitatively different relationships once this shared variance is removed. A further change was observed for the correlation between OCD and ADHD, where a modest negative correlation became more pronounced after controlling for *p*. This suggests that transdiagnostic genetic variance could mask greater divergence between these conditions. This pattern is consistent with clinical models positioning OCD and ADHD at opposing ends of the impulsivity–compulsivity continuum (Abramovitch et al., 2015; Hollander, 2005).

These changes in genetic relationships should be interpreted in light of how the transdiagnostic *p* factor was constructed. Because we modeled *p* as a single factor capturing genetic variance that was broadly shared across 11 psychiatric disorders, residual associations are conditional on removal of this shared component. This could have impacted disorders with higher loadings on the *p* factor, for example, MDD, more than others. Therefore, changes in the magnitude or direction of residual genetic correlations are not independent of the specifications of the model we conditioned on, even if results were robust across different models.

Relatedly, we acknowledge that latent factors derived from structural equation modeling do not represent causal entities (Van Loo et al., 2022) and are sensitive to which constructs are included in the model (Watts et al., 2020). Accordingly, the *p* factor should be interpreted as a data‐driven summary of genetic covariance rather than as a definitive or causal index of general psychopathology. Furthermore, our model of *p* captures genetic liability that is shared across all disorders included in the model, and remaining associations might reflect both disorder‐specific genetic effects and shared genetic liability among subsets of disorders (Grotzinger et al., 2022). As a result, disorder‐level genetic effects derived after removing *p* should be interpreted as conditional on the specified structure rather than as stable indicators of disorder‐specific genetic architecture. We retained a parsimonious common‐factor model because our primary aim was to approximate genetic variance shared broadly across disorders rather than to optimize global fit. However, in the absence of extensive sensitivity analyses across alternative specifications, *non‐p* findings should be viewed as hypothesis‐generating and interpreted with appropriate caution. Alternative specifications of transdiagnostic structure (Grotzinger et al., 2022) may yield different residual partitions, and future work can examine how these alternative formulations influence disorder‐specific genetic inferences.

Comparing genetic correlations between psychiatric disorders and external biobehavioural traits further demonstrated greater specificity after accounting for transdiagnostic effects, particularly for psychological and health‐related traits. Although many associations were attenuated, others strengthened or reversed in direction, revealing more differentiated genetic profiles for disorders such as MDD, ANX, SCZ and BIP. These findings suggest that transdiagnostic genetic risk can obscure disorder‐specific relationships with external traits, with implications for prediction and etiological inference. More broadly, recent research has shown widespread genetic correlations observed across psychiatric and physical health conditions (Brandt et al., 2023; Lawrence et al., 2024), highlighting the importance of distinguishing shared from disorder‐specific components when interpreting such associations.

Together, our findings highlight how isolating transdiagnostic genetic risk provides novel insight into disorder‐specific genetic architecture and a more nuanced understanding of their comorbidities and co‐occurrences with psychological and health‐related traits. Consequently, our work emphasizes the significance of considering specificity as well as generality in psychiatric genetics. By demonstrating distinct genetic correlations and outcomes associated with psychiatric conditions independent of transdiagnostic effects, our findings pave the way for new avenues of research. One such application is the use of *non‐p* summary statistics to construct polygenic scores to predict greater disorder specificity (Keser et al., 2026), or to investigate specificity in the associations between psychiatric disorders and cognitive and behavioral dimensions (Liao et al., 2026). This is likely to provide further insight into how genetic risk unfolds over the lifespan and might offer new opportunities to examine gene‐environment interplay.

### Limitations

Our findings should be interpreted in the context of several limitations. First, this work is necessarily grounded in case‐control GWASs based on traditional diagnoses perfused with transdiagnostic effects. This highlights the need for genetic studies using more empirically derived dimensional phenotypes, such as those proposed by HiTOP (Kotov et al., 2017) and RDoC (Insel et al., 2010). Second, our study is subject to limitations common to GWAS research. For example, the underlying GWASs are limited to individuals of European ancestry, limiting generalizability of findings to other populations. Additionally, the contributing GWASs are meta‐analyses of different cohorts that may be subject to heterogeneity that cannot be fully quantified. More broadly, our findings may be influenced by cross‐trait assortative mating (Border et al., 2022) and population stratification (Novembre et al., 2008).

Despite these limitations, separating shared and disorder‐specific genetic influences offers a useful framework for advancing psychiatric genetics. While the p factor is valuable for understanding general genetic influences, isolating the transdiagnostic effects captured by p may be useful in sharpening research on specific genetic influences, particularly in the context of developmental psychopathology and clinical epidemiological studies. Genetic effects that are disorder‐specific might inform future research into causes and consequences of psychiatric conditions applying causal designs including mendelian randomization and longitudinal models (Allegrini et al., 2022; Pingault et al., 2017).

## CONCLUSION

In conclusion, our results show that isolating transdiagnostic effects from major psychiatric disorders provides novel insight into disorder‐specific genetic architecture and a more precise understanding of the comorbidities and co‐occurrences in psychopathology. Until better correspondence between psychiatric diagnoses and the genetic architecture of psychopathology is achieved, isolating *p* from diagnostic categories can sharpen genetic research by focusing on disorder‐specific genetic effects.

## AUTHOR CONTRIBUTIONS

**Engin Keser**: Conceptualization; investigation; methodology; writing—review and editing; visualization; writing—original draft; validation; software; formal analysis; project administration. **Wangjingyi Liao**: Conceptualization; investigation; methodology; writing—review and editing; visualization; writing—original draft; validation; software; formal analysis; project administration. **Andrea G. Allegrini**: Writing—review and editing; supervision; conceptualization. **Kaili Rimfeld**: Writing—review and editing; supervision. **Thalia C. Eley**: Writing—review and editing. **Robert Plomin**: Conceptualization; writing—original draft; supervision; methodology; writing—review and editing. **Margherita Malanchini**: Conceptualization; writing—original draft; supervision; methodology; writing—review and editing; visualization.

## CONFLICT OF INTEREST STATEMENT

The authors declare no conflicts of interest.

## ETHICAL CONSIDERATIONS

Not applicable. This study did not involve data collection. All analyses were conducted using publicly available GWAS summary statistics for which ethical approval and participant consent were obtained by the original studies.

## Supporting information

Supporting Information S1

Supporting Information S2

## Data Availability

The data that support the findings of this study are available in Psychiatric Genomics Consortium at https://pgc.unc.edu. These data were derived from the following resources available in the public domain—All GWAS, https://pgc.unc.edu/for‐researchers/download‐results/.
