# Supplementary material for: Isolating transdiagnostic effects reveals specific genetic profiles in psychiatric disorders
Source: JCPP Adv. 2026 May 2:e70129. Online ahead of print. doi: 10.1002/jcv2.70129 (PMC13338967; doi:10.1002/jcv2.70129)
Supplement: Supplementary file 1 — Supporting Information S1 [file JCV2-9999-e70129-s001.docx]

Isolating transdiagnostic effects reveals specific genetic profiles in

psychiatric disorders

Supporting Information

**Supporting Notes**

| Appendix S1. Extensions of preregistered analyses …………………………………………………… | 3 |
| --- | --- |
| Appendix S2. Genomic structural equation modelling ………………………………………………. | 3 |
| Processing of summary statistics ………………………………………………………………………….. | 3 |
| Effective sample size calculation …………………………………………………………………………… | 4 |
| Appendix S3. An alternative approach to identify genetic variants associated with psychiatric disorders after accounting for transdiagnostic effects using GWAS-by-subtraction ……………………………………………………………………………………………………………….. | 5 |
| Figure S1. Schematic overview of the GWAS-by-subtraction approach for creating a latent residual for the schizophrenia-specific factor ………………………………………………. | 6 |
| Appendix S4. A common factor model to capture transdiagnostic effects ………………… | 6 |

**Supporting Figures**

| Figure S2. Manhattan plot of the ANX *non-p* GWAS …………………………………………………. | 7 |
| --- | --- |
| Figure S3. Manhattan plot of the MDD *non-p* GWAS ………………………………………………… | 8 |
| Figure S4. Manhattan plot of the PTSD *non-p* GWAS ………………………………………………… | 9 |
| Figure S5. Manhattan plot of the BIP *non-p* GWAS ……………………………………………………. | 10 |
| Figure S6. Manhattan plot of the SCZ *non-p* GWAS …………………………………………………… | 11 |
| Figure S7. Manhattan plot of the ADHD *non-p* GWAS ……………………………………………….. | 12 |
| Figure S8. Manhattan plot of the ASD *non-p* GWAS ………………………………………………….. | 13 |
| Figure S9. Manhattan plot of the ALCH *non-p* GWAS ………………………………………………. | 14 |
| Figure S10. Manhattan plot of the OCD *non-p* GWAS ……………………………………………….. | 15 |
| Figure S11. Manhattan plot of the AN *non-p* GWAS ………………………………………………….. | 16 |
| Figure S12. Manhattan plot of the TS *non-p* GWAS …………………………………………………… | 17 |
| Figure S13. The tissue type enrichment results on 53 specific tissue types by GTEx of MDD uncorrected and corrected for *p* ………………………………………………………………………. | 18 |
| Figure S14. The brain sample enrichment results on 11 general brain developmental stages by BrainSpan of MDD uncorrected and corrected for *p* ………………………………….. | 19 |
| Figure S15. The tissue type enrichment results on 53 specific tissue types by GTEx of BIP uncorrected and corrected for *p* …………………………………………………………………………. | 20 |
| Figure S16. The brain sample enrichment results on 11 general brain developmental stages by BrainSpan of BIP uncorrected and corrected for *p* ……………………………………. | 21 |
| Figure S17. The tissue type enrichment results on 53 specific tissue types by GTEx of Schizophrenia uncorrected and corrected for *p* ………………………………………………………… | 22 |
| Figure S18. The brain sample enrichment results on 11 general brain developmental stages by BrainSpan of Schizophrenia uncorrected and corrected for *p* ……………………. | 23 |
| Figure S19. The tissue type enrichment results on 53 specific tissue types by GTEx of ADHD uncorrected and corrected for *p* …………………………………………………………………….. | 24 |
| Figure S20. The brain sample enrichment results on 11 general brain developmental stages by BrainSpan of ADHD uncorrected and corrected for *p* ………………………………… | 25 |
| Figure S21. The tissue type enrichment results on 53 specific tissue types by GTEx of ASD uncorrected and corrected for *p* ………………………………………………………………………… | 26 |
| Figure S22. The brain sample enrichment results on 11 general brain developmental stages by BrainSpan of ASD uncorrected and corrected for *p* ……………………………………. | 27 |
| Figure S23. The tissue type enrichment results on 53 specific tissue types by GTEx of ALCH uncorrected and corrected for *p* ……………………………………………………………………… | 28 |
| Figure S24. The brain sample enrichment results on 11 general brain developmental stages by BrainSpan of ALCH uncorrected and corrected for *p* …………………………………. | 29 |
| Figure S25. The tissue type enrichment results on 53 specific tissue types by GTEx of AN uncorrected and corrected for *p* …………………………………………………………………………. | 30 |
| Figure S26. The brain sample enrichment results on 11 general brain developmental stages by BrainSpan of AN uncorrected and corrected for *p* …………………………………….. | 31 |
| Figure S27. Genetic correlations between psychiatric disorders before and after accounting for transdiagnostic effects obtained using the GWAS-by-subtraction modelling approach ………………………………………………………………………………………………….. | 32 |
| Figure S28. Genetic correlations between the psychiatric disorders and anthropometric traits before and after controlling for *p* …………………………………………… | 33 |
| Figure S29. Genetic correlations between psychiatric disorders and socio-demographic traits before and after controlling for *p* ……………………………………………….. | 34 |

# **Appendix S1: Extensions of pre-registered analyses**

We extended our pre-registered analytical plan by incorporating an alternative approach to isolating transdiagnostic genetic effects from each psychiatric disorder. This approach, based on a single common factor model, is presented in the main manuscript, whereas the modelling approach specified in our pre-registration is presented in Appendix S3. Results were highly consistent across both modelling frameworks.

# **Appendix S2: Genomic Structural Equation Modelling**

Genomic structural equation modelling (Genomic SEM; Grotzinger et al., 2019) is a statistical framework that applies structural equation modelling techniques to genome-wide association study (GWAS) summary statistics to model the genetic covariance structure among complex traits.

Genomic SEM uses a two-stage estimation process. In the first stage, the genetic covariance matrix and its associated sampling covariance matrix are estimated. In the second stage, a model is specified, and model parameters are estimated by minimizing the discrepancy between the model-implied and empirical covariance matrices. Model fit is evaluated using standard indices, including standardized root mean square residual (SRMR), model *χ*2, Akaike Information Criterion (AIC) and the Comparative Fit Index (CFI).

## *Processing of summary statistics*

GWAS summary statistics were processed for use in Genomic SEM as follows. Standard quality control (QC) filters were applied to all GWAS summary statistics using the *munge* function in Genomic SEM. Munging summary statistics reduces the number of SNPs to HapMap3 SNPs and applies pruning based on minor allele frequency (MAF) < 0.01 and imputation score (INFO) < 0.9, when this information is available. The “munged” summary statistics were then used as input for the multivariable version of linkage disequilibrium score regression (LDSC) implemented within Genomic SEM. LD weights used to estimate the LDSC regression model were obtained from the 1000 Genome Phase 3 European LD scores, excluding the major histocompatibility complex (MHC) region due to complex LD structures in this region that can bias estimates.

LDSC produces two output matrices: the genetic covariance matrix and the sampling covariance matrix. The genetic covariance matrix contains SNP-based heritability estimates on the diagonal and genetic covariances on the off-diagonal. Because all the disorders (except problem alcohol use) are binary, estimates were converted to the liability scale using the population prevalences in the original publications. For meta-analysed GWAS summary statistics, the sum of effective sample sizes across contributing cohorts was used. The sampling covariance matrix contains squared standard errors (e.g., the sampling variances) on the diagonal and sampling covariances on the off-diagonal, reflecting sampling dependencies that may arise from sample overlap across included traits.

Next, the *sumstats* function in Genomic SEM was used to standardize SNPs across traits. Summary statistics for each of the 11 disorders were restricted to SNPs with MAF > 1% and INFO > 0.6, and to SNPs that were present for all the 11 disorders. SNPs were further limited to those present in the European-only 1000 Genomes Phase 3 reference panel. After these QC steps, 3,746,806 SNPs were retained across all 11 disorders.

We estimated the common factor model using the default weighted least squares (WLS) estimator implemented in Genomic SEM. This WLS-estimated model was used for all analyses reported in the manuscript. As a sensitivity analysis, we re-estimated the same model using the maximum likelihood (ML) estimator. The ML solution produced a similar pattern of factor loadings, but poorer overall model fit; therefore, WLS solution was retained for all primary analyses.

## *Effective sample size calculation*

We calculated effective sample sizes for each of the 11 disorders following the method described by Grotzinger et al. (2023). According to this method, the effective sample size for GWAS that was meta-analysed was calculated by summing the effective sample sizes across contributing cohorts. This approach incorporates cohort-specific ascertainment, which provided an unbiased estimate of liability scale heritability for binary traits. We refer the reader to Grotzinger et al. (2023) for a detailed explanation of why this method produces a more accurate estimate of heritability for binary traits.

# **Appendix S3: Alternative approach to identify genetic variants associated with psychiatric disorders after accounting for transdiagnostic effects using GWAS-by-subtraction.**

We implemented a two-stage modelling strategy. In the first stage, we constructed a genomic *p* factor using a common factor model in Genomic SEM (see Supplementary Notes 2). In the second stage, we applied GWAS-by-subtraction (Demange et al., 2021) framework to separate genetic effects associated with the genomic p factor from those associated with each psychiatric disorder. This approach allowed us to identify genetic effects associated with each disorder independent of transdiagnostic genetic effects. Figure S1 illustrates the GWAS-by-subtraction model using schizophrenia (SCZ) as an example. The process was repeated for each of the 11 disorders to isolate residual (*non-p*) genetic variance associated with each condition.

GWAS-by-subtraction (Demange et al., 2021) is a model implemented within Genomic SEM that estimates, for each SNP, its effect on a trait independent of its effect on another trait. Using SCZ as an example, GWAS summary statistics for *p* factor and SCZ were regressed on a latent factor, ‘*p* SCZ’, which represents genetic variance that is shared across the 11 disorders (Figure S1, left). SCZ was further regressed on a second latent factor representing the residual genetic variance in SCZ left after regressing out variance related to p, i.e., *non-p* SCZ (Figure S1, right). By construction, the *non-p* SCZ factor is independent of genetic variance in the p-factor (*r*_g_ = 0). In other words, the *non-p* SCZ factor represents genetic variance in SCZ that is not accounted for by the p factor. The two latent variables, *p* SCZ and *non-p* SCZ are then regressed on each SNP, iterating across all SNPs in the GWAS, resulting in new GWAS summary statistics for *non-p* SCZ.

The results obtained using this alternative approach were highly consistent with those reported in the main manuscript, as reflected by the patterns of changes in the genetic correlations shown in Figure S28.


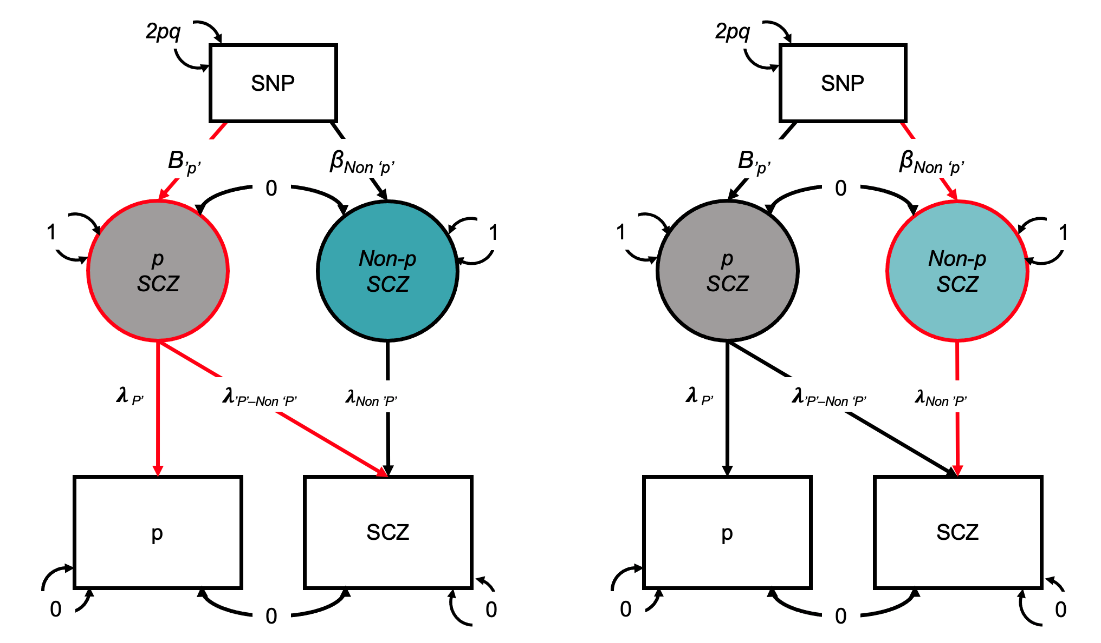


**Figure S1.** **Schematic overview of the GWAS-by-subtraction approach for creating a latent residual for the schizophrenia (SCZ)-specific factor (Figure adapted from Demange et al., 2021).** In this diagram, squares represent the observed SNP and the GWAS of p-factor and SCZ. Circles represent the latent (unobserved) variables, i.e., *p* SCZ and *Non-p* SCZ. Single-headed arrows represent linear regression associations pointing from the independent variable to the dependent variable. Two-headed arrows represent covariance relationships. The covariances between *p* factor and SCZ and between *p* SCZ and *Non-p* SCZ are fixed to 0. The variance of the SNP is fixed to the value of 2pq (p= reference allele frequency, q= alternative allele frequency, based on 1000 Genomes Project phase 3). The residual variances of *p* and SCZ are fixed to 0, so that all variance is explained by the latent factors. The variances of the latent factors are fixed to 1. λ= freely estimated factor loadings that are equivalent to regression weights. Β= regression effects of *p* SCZ and *Non-p* SCZ on the SNP.

We calculated the expected sample size of the latent non-p factors following the method described by Demange et al. (2021). According to this method, effective sample sizes, estimated based on the formula described by Mallard et al. (2022), are adjusted by multiplying them by the residual heritability (squared unstandardized path loading).

## Appendix S4: A common factor model to capture transdiagnostic effects

To capture transdiagnostic genetic effects across all 11 disorders, we fitted a common factor model to the genetic covariance matrix in Genomic SEM. In this model, all disorders loaded on a single latent factor. The model yielded adequate fit: χ2(44) = 950.4836, AIC = 994.48, CFI = .82, SRMR = .12. For sensitivity analysis, the same model was re-estimated using the ML estimator, which showed poorer overall fit (χ2(44) = 1245.822, AIC = 1289.82, CFI = .77, SRMR = .13).

# **SUPPLEMENTARY FIGURES**


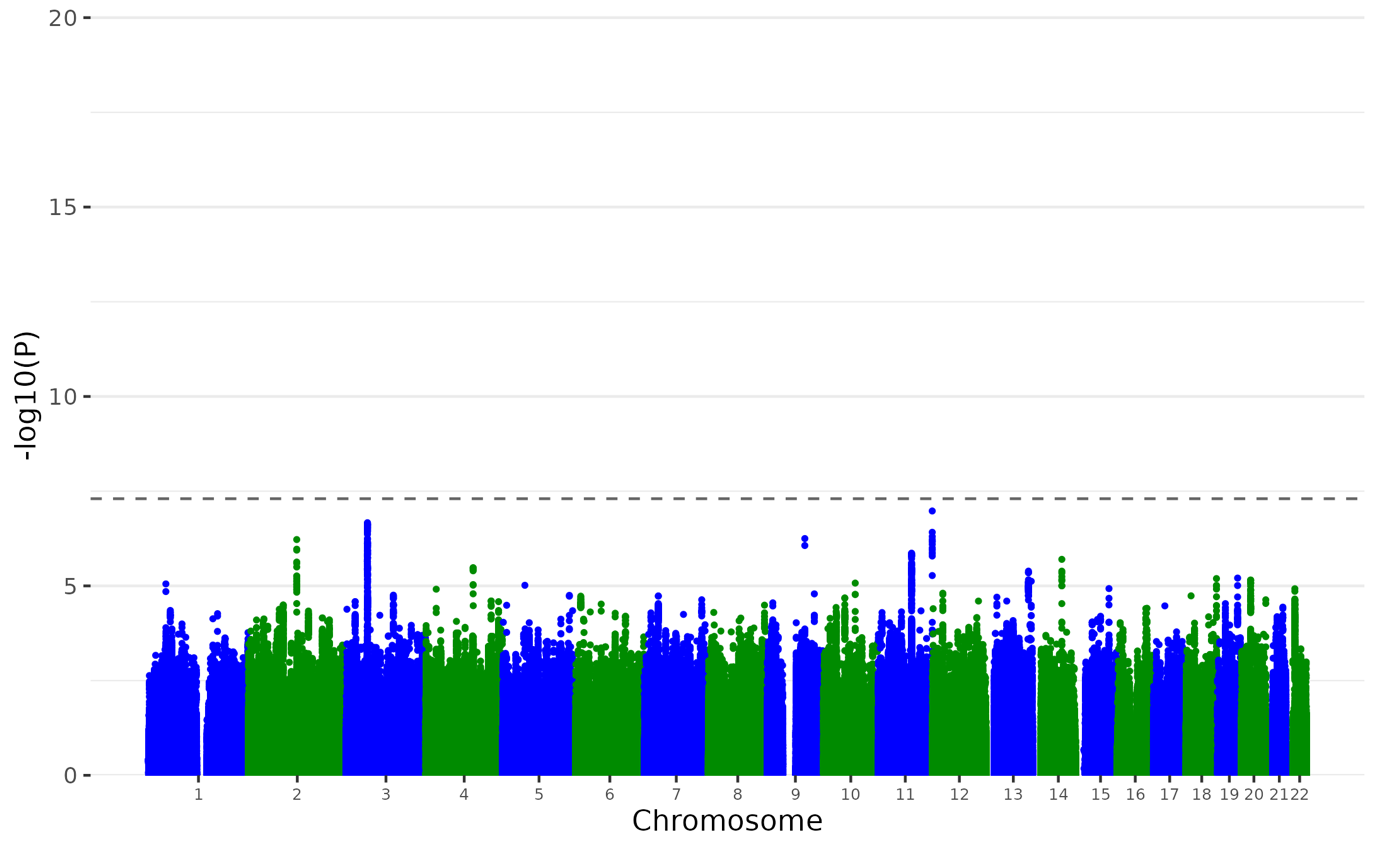


**Figure S2. Manhattan plot of the ANX *non-p* GWAS.** Plot of the -log_10_(p-value) associated with the Wald test (two-sided) of β_p_ for all SNPs ordered by chromosome and base position.


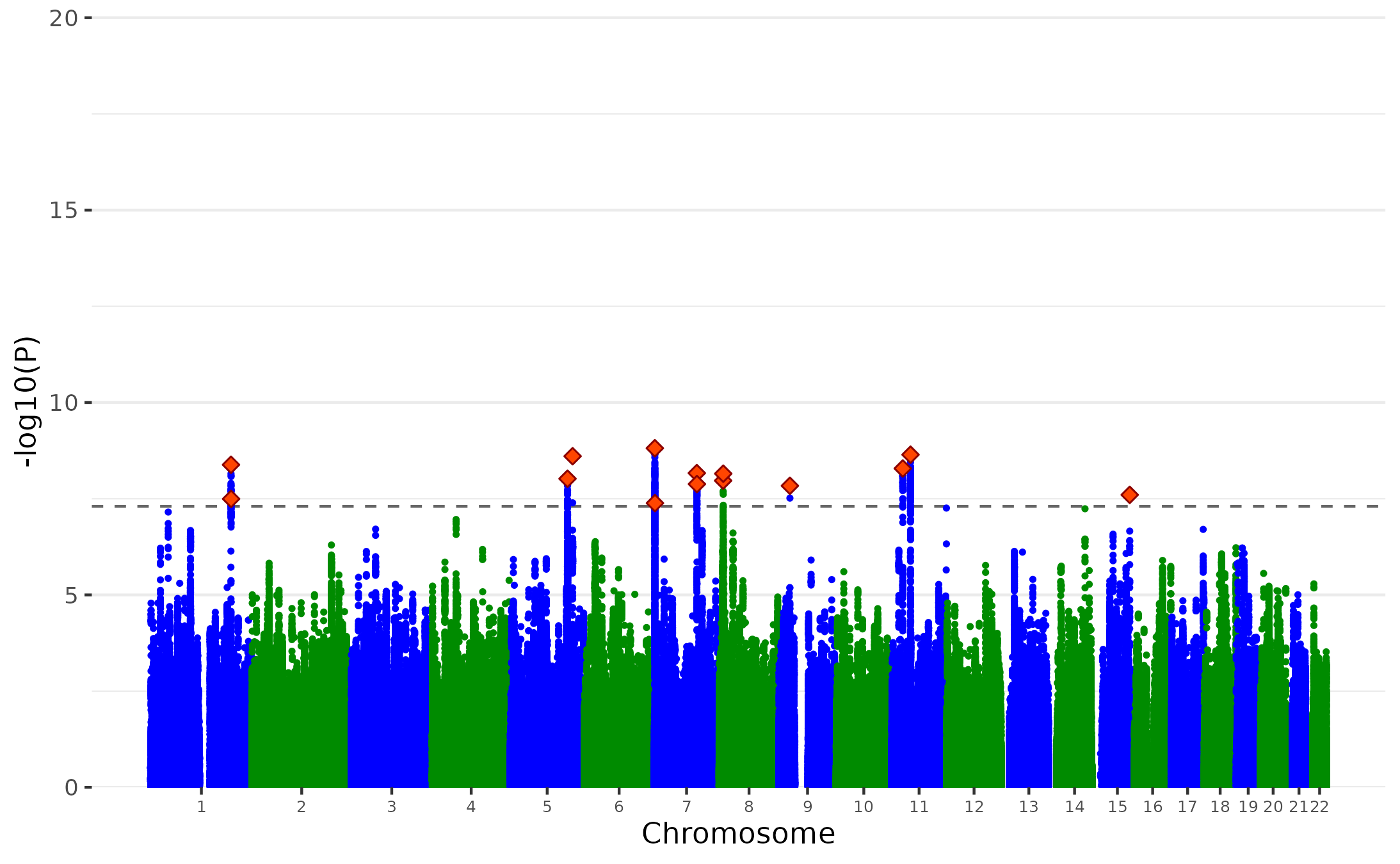


**Figure S3. Manhattan plot of the MDD *non-p* GWAS.** Plot of the -log_10_(p-value) associated with the Wald test (two-sided) of β_p_ for all SNPs ordered by chromosome and base position. Red diamonds indicate genome-wide significant independent hits (within a 250Kb window and r^2^ < .1) associations.


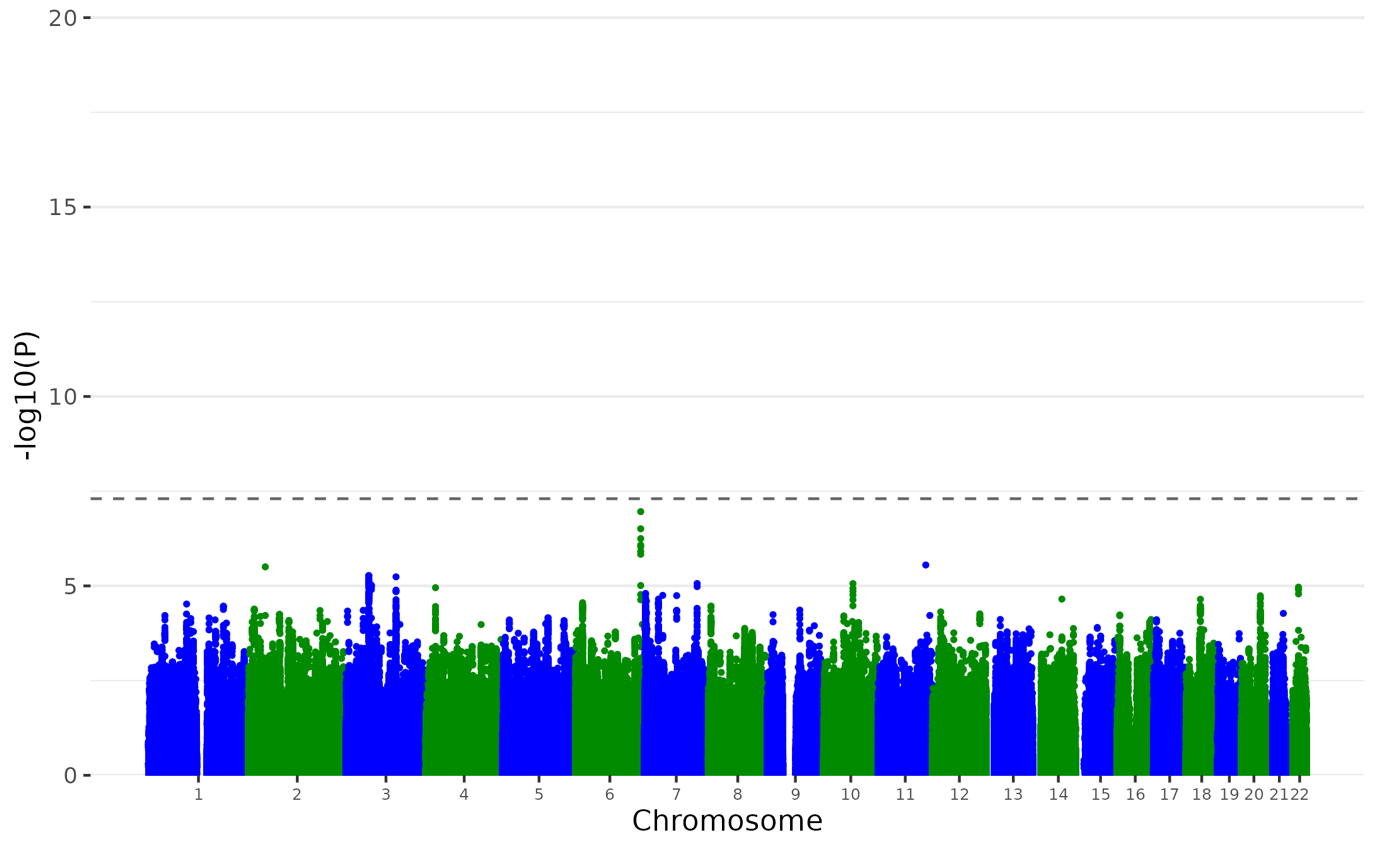


**Figure S4. Manhattan plot of the PTSD *non-p* GWAS.** Plot of the -log_10_(p-value) associated with the Wald test (two-sided) of β_p_ for all SNPs ordered by chromosome and base position.


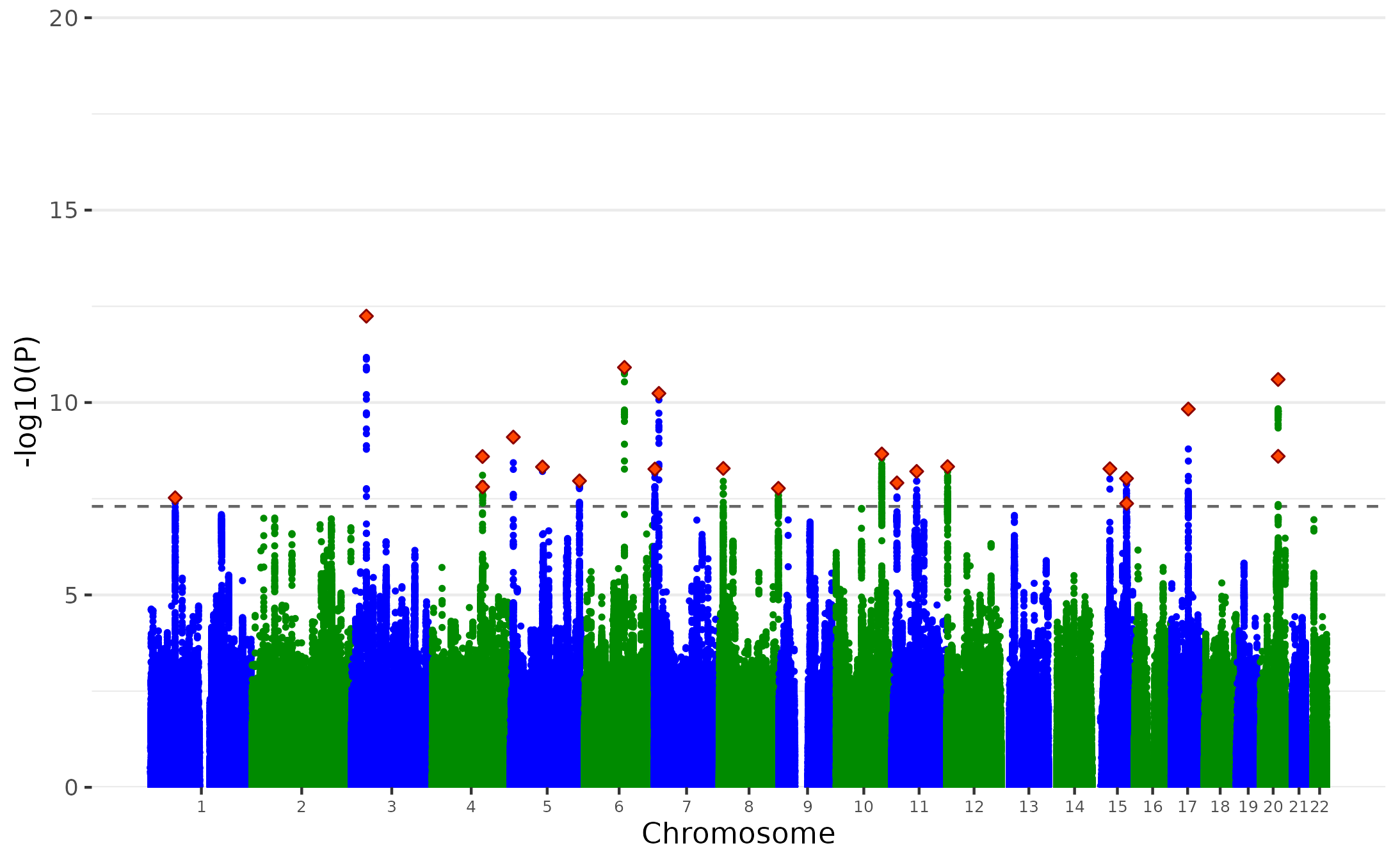


**Figure S5. Manhattan plot of the BIP *non-p* GWAS.** Plot of the -log_10_(p-value) associated with the Wald test (two-sided) of β_p_ for all SNPs ordered by chromosome and base position. Red diamonds indicate genome-wide significant independent hits (within a 250Kb window and r^2^ < .1) associations.


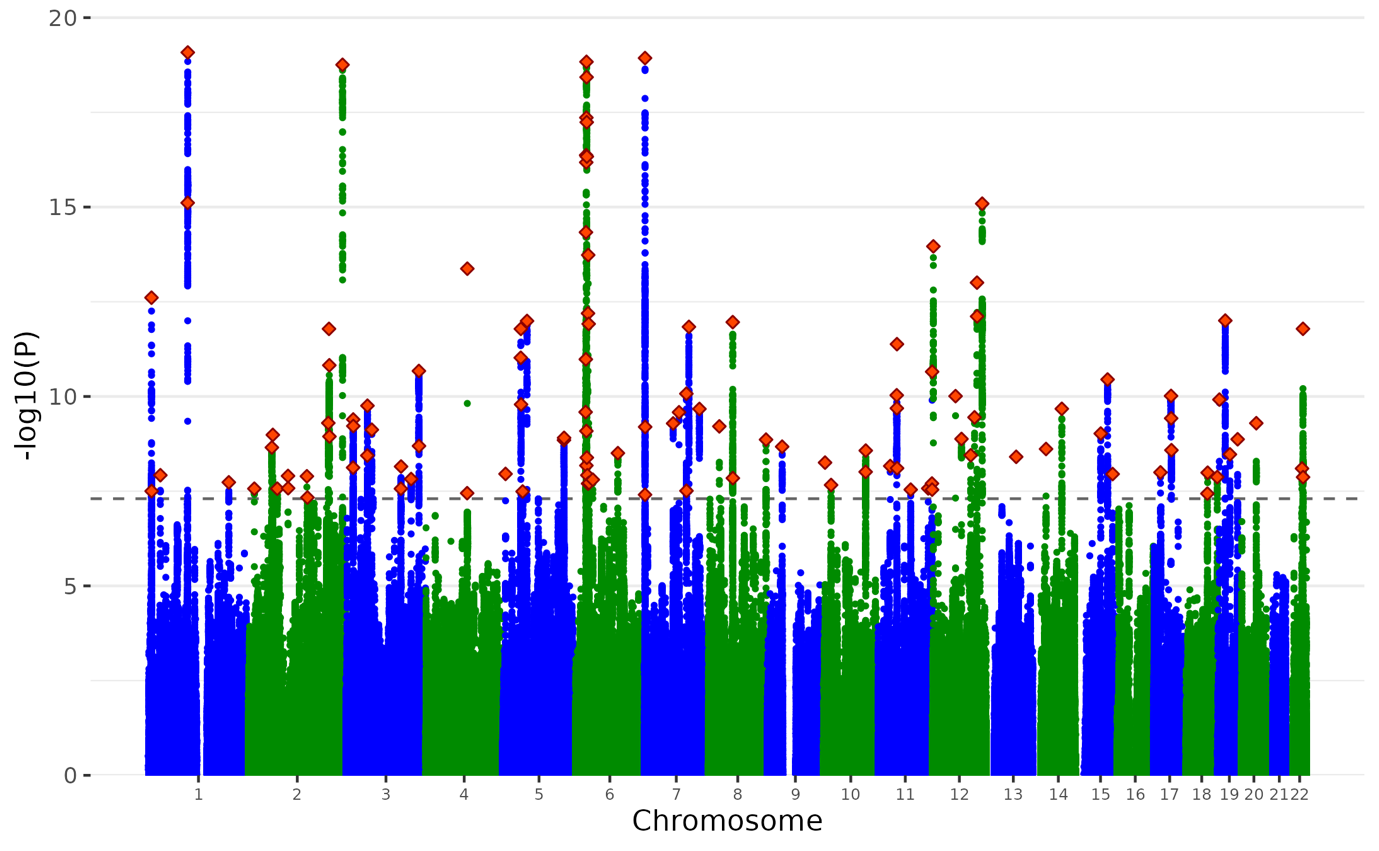


**Figure S6. Manhattan plot of the SCZ *non-p* GWAS.** Plot of the -log_10_(p-value) associated with the Wald test (two-sided) of β_p_ for all SNPs ordered by chromosome and base position. Red diamonds indicate genome-wide significant independent hits (within a 250Kb window and r^2^ < .1) associations.


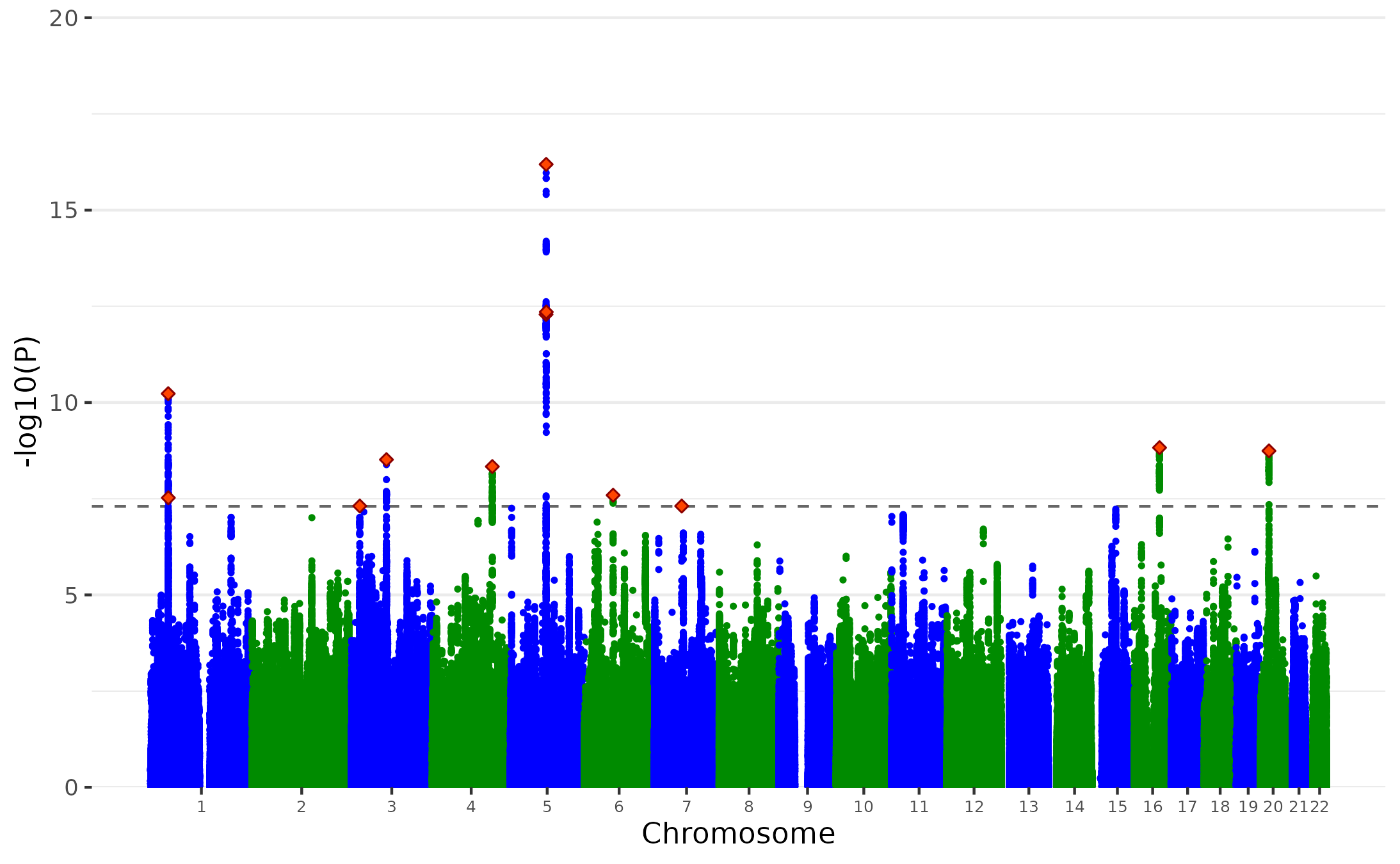


**Figure S7. Manhattan plot of the ADHD *non-p* GWAS.** Plot of the -log_10_(p-value) associated with the Wald test (two-sided) of β_p_ for all SNPs ordered by chromosome and base position. Red diamonds indicate genome-wide significant independent hits (within a 250Kb window and r^2^ < .1) associations.


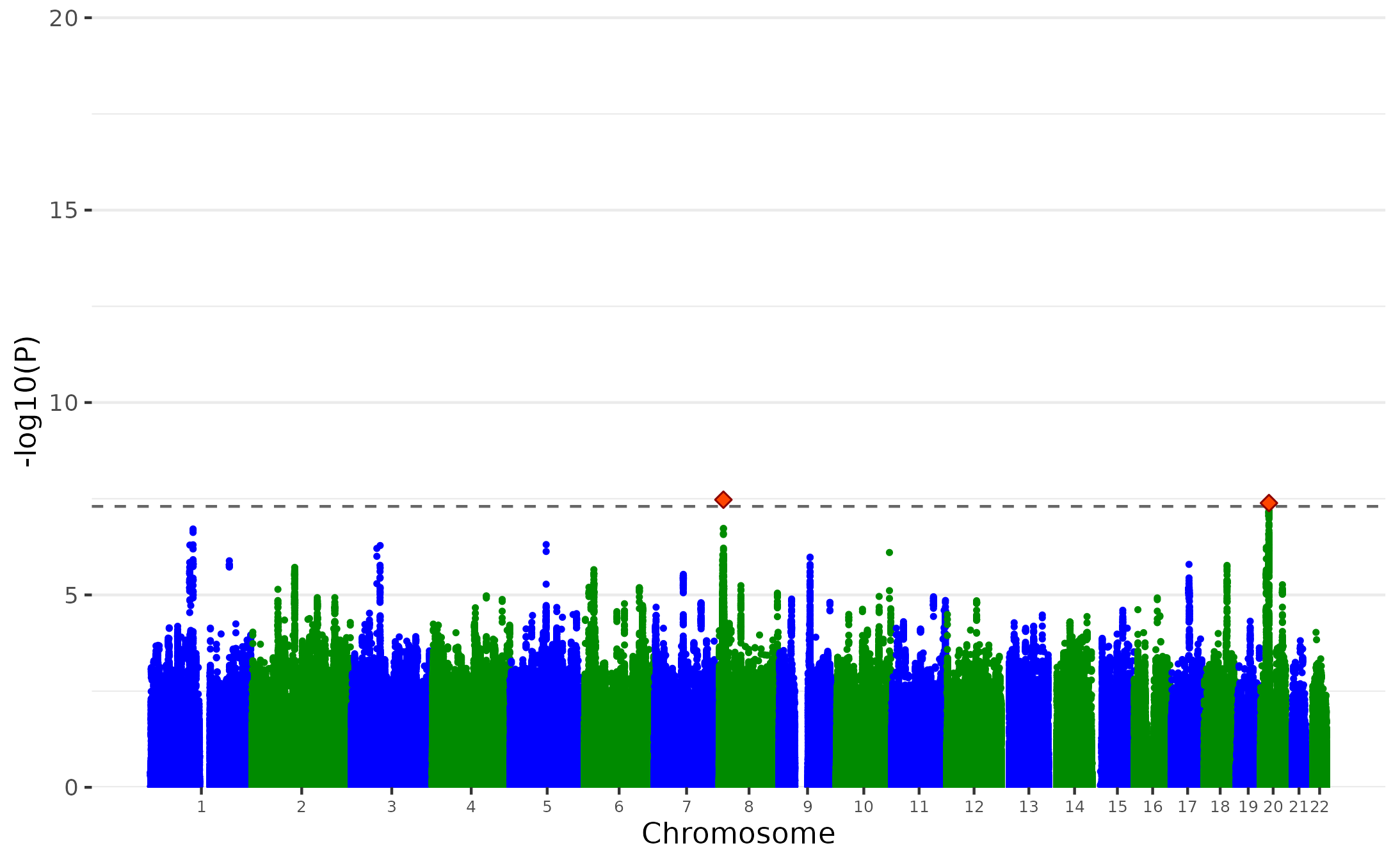


**Figure S8. Manhattan plot of the ASD *non-p* GWAS.** Plot of the -log_10_(p-value) associated with the Wald test (two-sided) of β_p_ for all SNPs ordered by chromosome and base position. Red diamonds indicate genome-wide significant independent hits (within a 250Kb window and r^2^ < .1) associations.


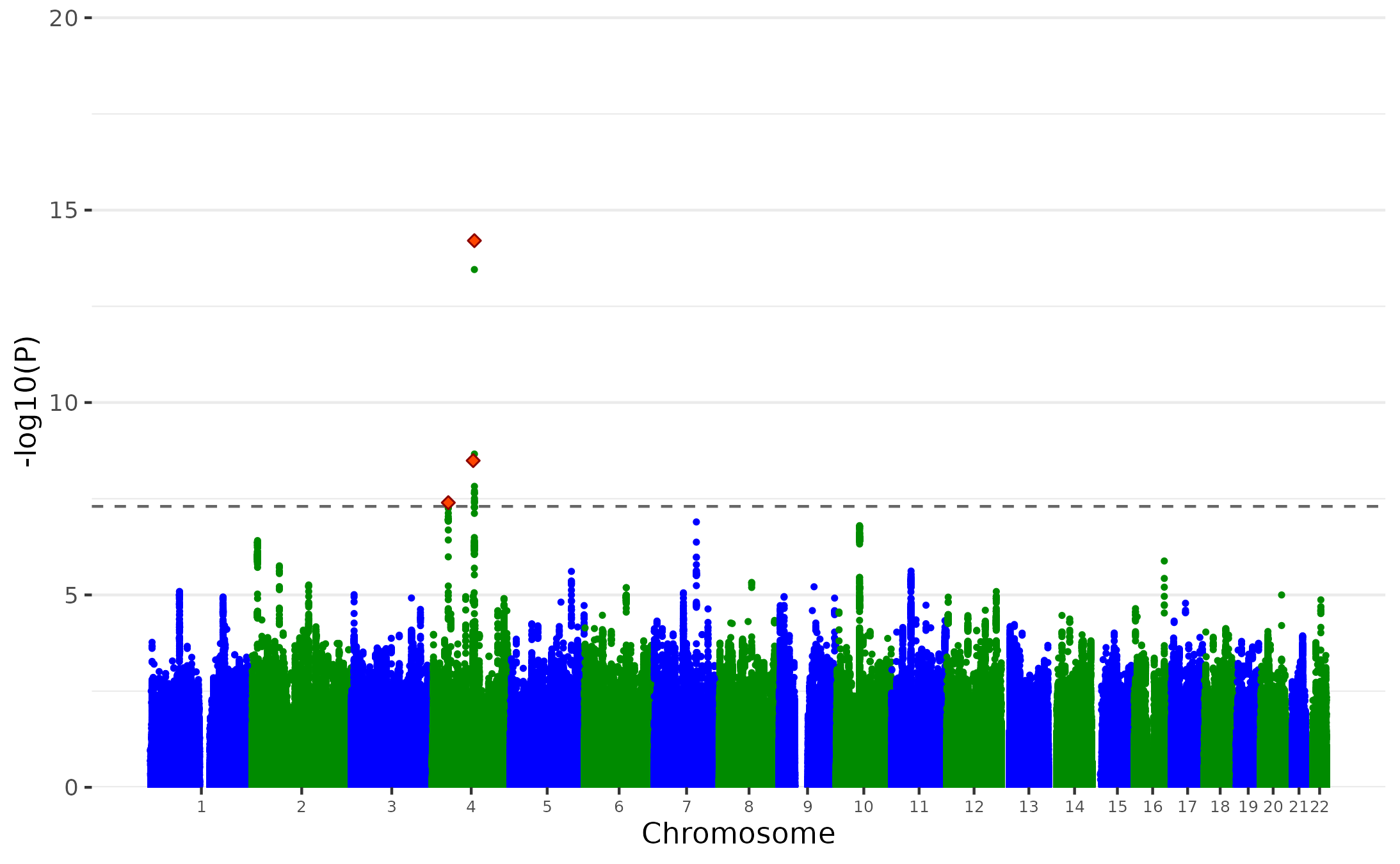


**Figure S9. Manhattan plot of the ALCH *non-p* GWAS.** Plot of the -log_10_(p-value) associated with the Wald test (two-sided) of β_p_ for all SNPs ordered by chromosome and base position. Red diamonds indicate genome-wide significant independent hits (within a 250Kb window and r^2^ < .1) associations.


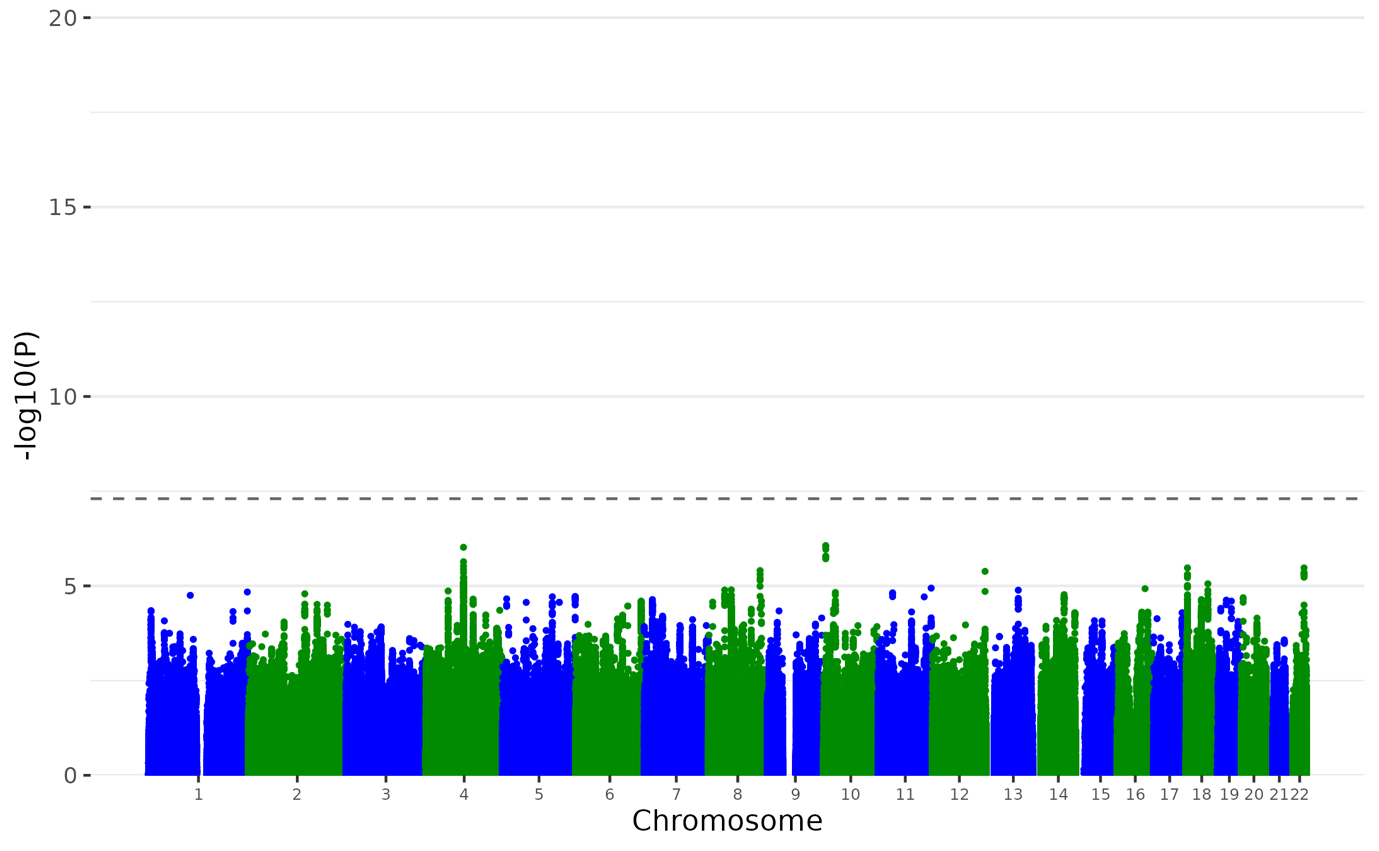


**Figure S10. Manhattan plot of the OCD *non-p* GWAS.** Plot of the -log_10_(p-value) associated with the Wald test (two-sided) of β_p_ for all SNPs ordered by chromosome and base position.


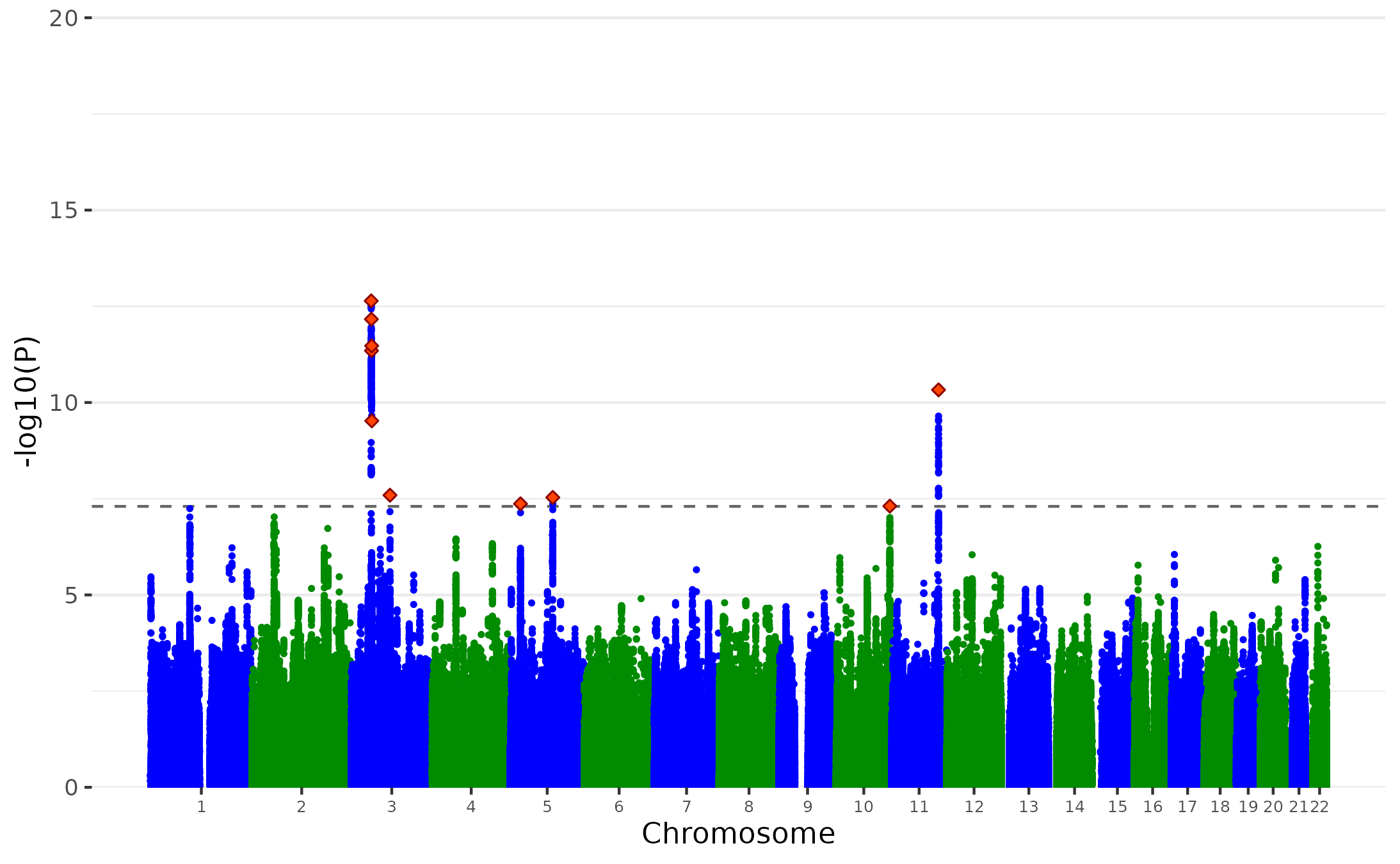


**Figure S11. Manhattan plot of the AN *non-p* GWAS.** Plot of the -log_10_(p-value) associated with the Wald test (two-sided) of β_p_ for all SNPs ordered by chromosome and base position. Red diamonds indicate genome-wide significant independent hits (within a 250Kb window and r^2^ < .1) associations.


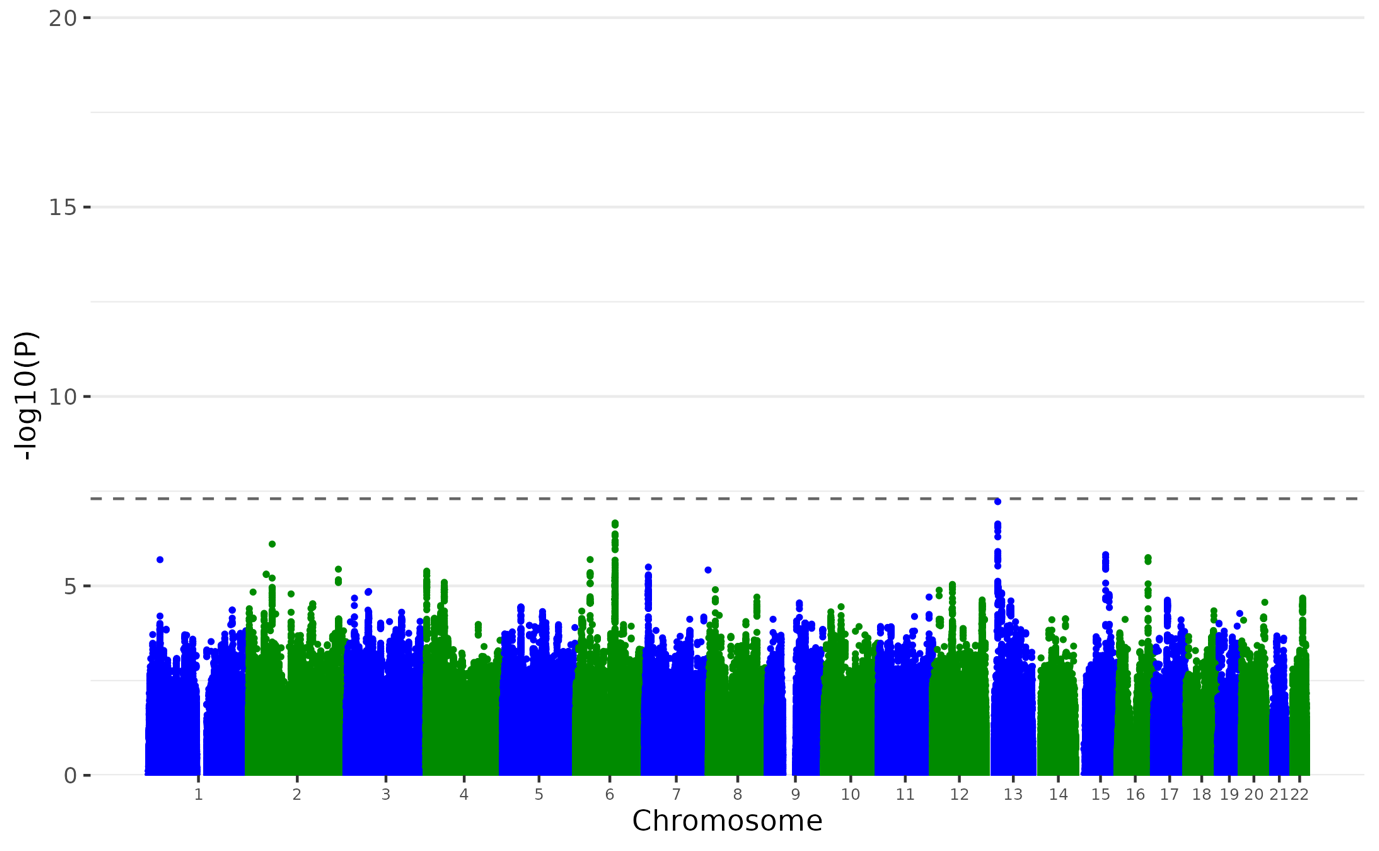


**Figure S12. Manhattan plot of the TS *non-p* GWAS.** Plot of the -log_10_(p-value) associated with the Wald test (two-sided) of β_p_ for all SNPs ordered by chromosome and base position.

MDD uncorrected for p


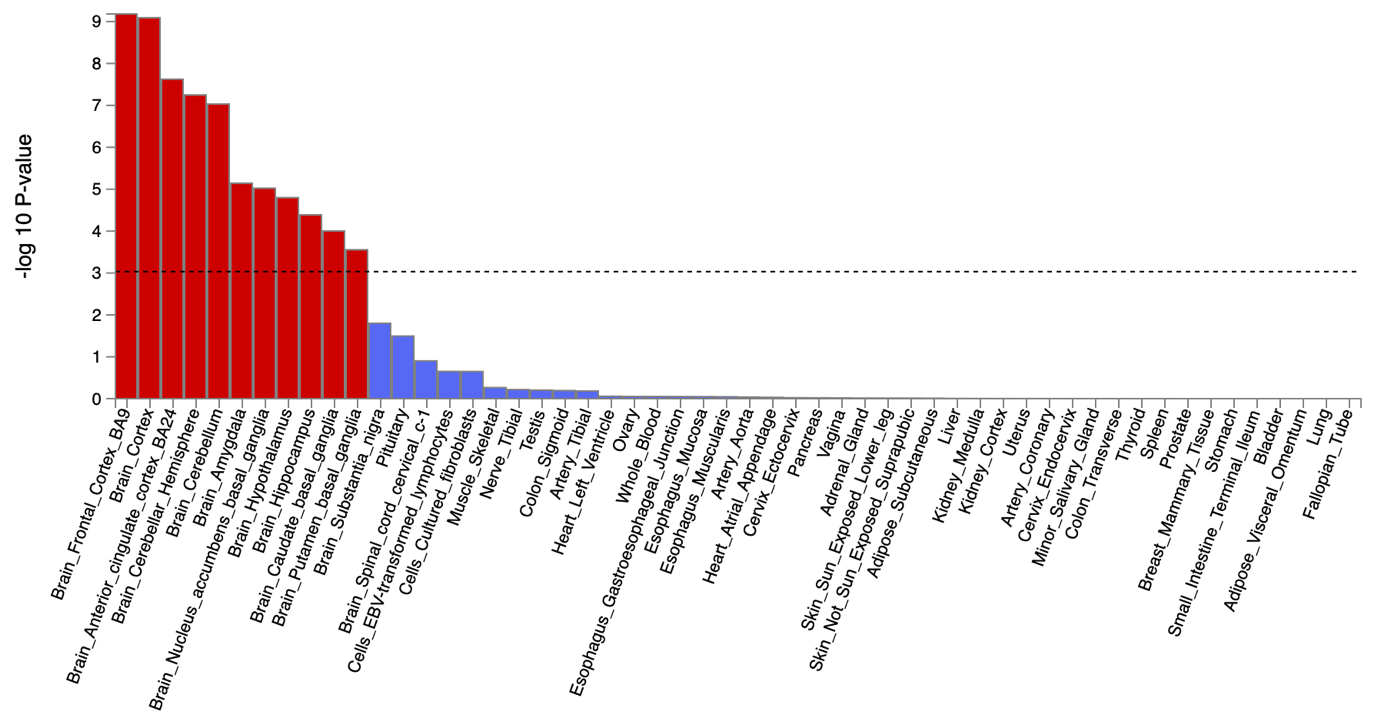


MDD corrected for p


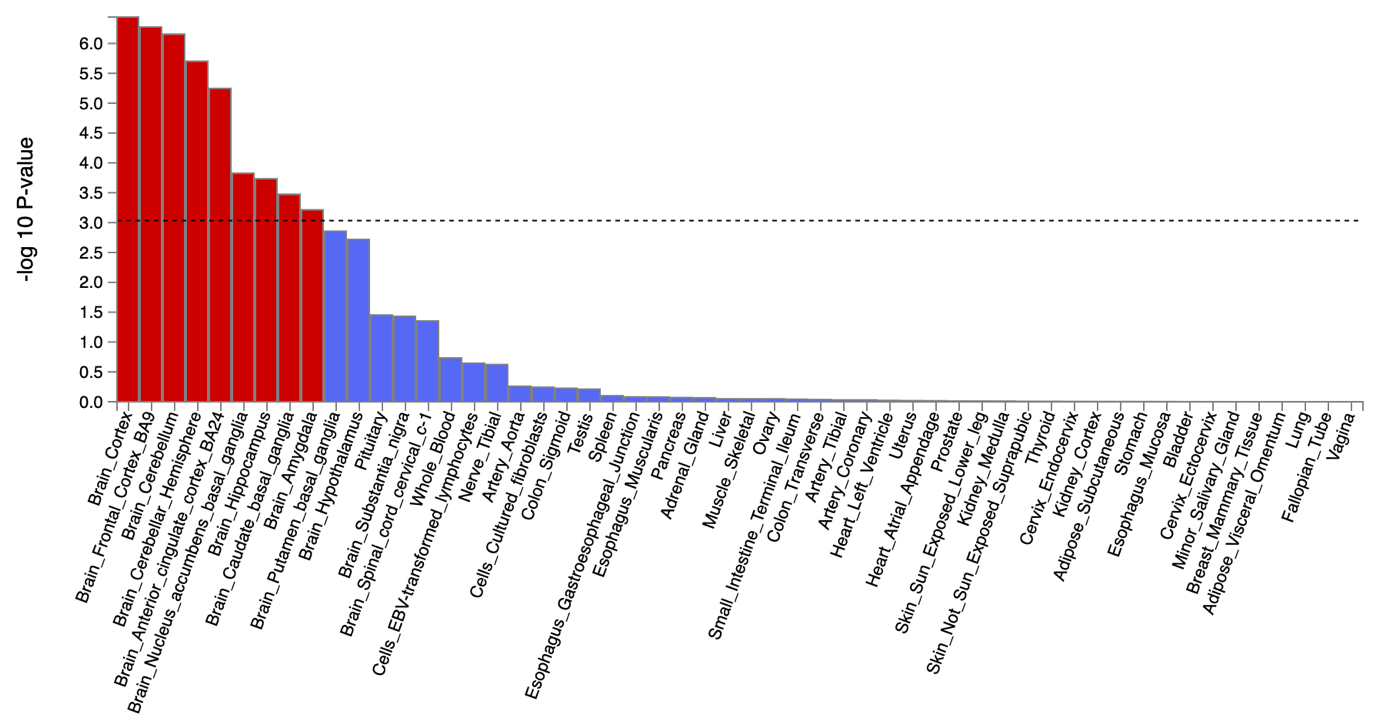


**Figure S13. The tissue type enrichment results on 53 specific tissue types by GTEx of MDD uncorrected and corrected for *p***

Results (-log10 (one-sided P-value)) from MAGMA gene-property analysis of relationships between tissue specific gene expression profiles (GTEx v.7) and MDD associations before and after correcting for *p*. The test was performed for average gene-expression per tissue type conditioning on the average expression across all categories. Dotted lines indicate significant results after Bonferroni correction. The full results are available in Supplementary Tables 28-31.


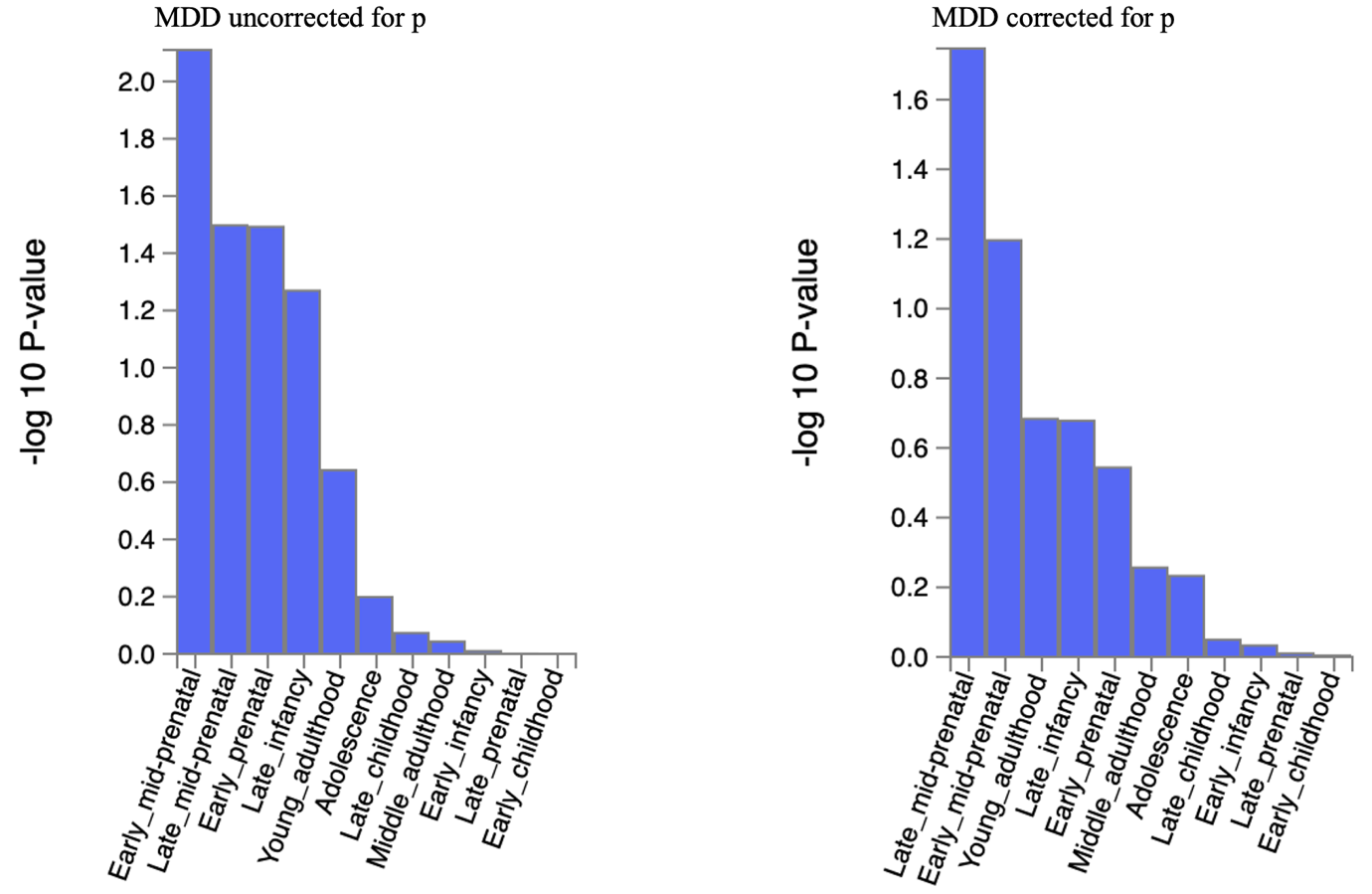


**Figure S14. The brain sample enrichment results on 11 general brain developmental stages by BrainSpan of MDD uncorrected and corrected for *p***

Results (-log10 (one-sided P-value)) from MAGMA gene-property analysis of relationships between gene expression data of developmental brain samples (BrainSpan) and MDD associations before and after correcting for p. The test was performed for average gene-expression per brain sample conditioning on the average expression across all developmental stages. Dotted lines indicate significant results after Bonferroni correction. The full results are available in Supplementary Tables 28-31.

BIP uncorrected for p


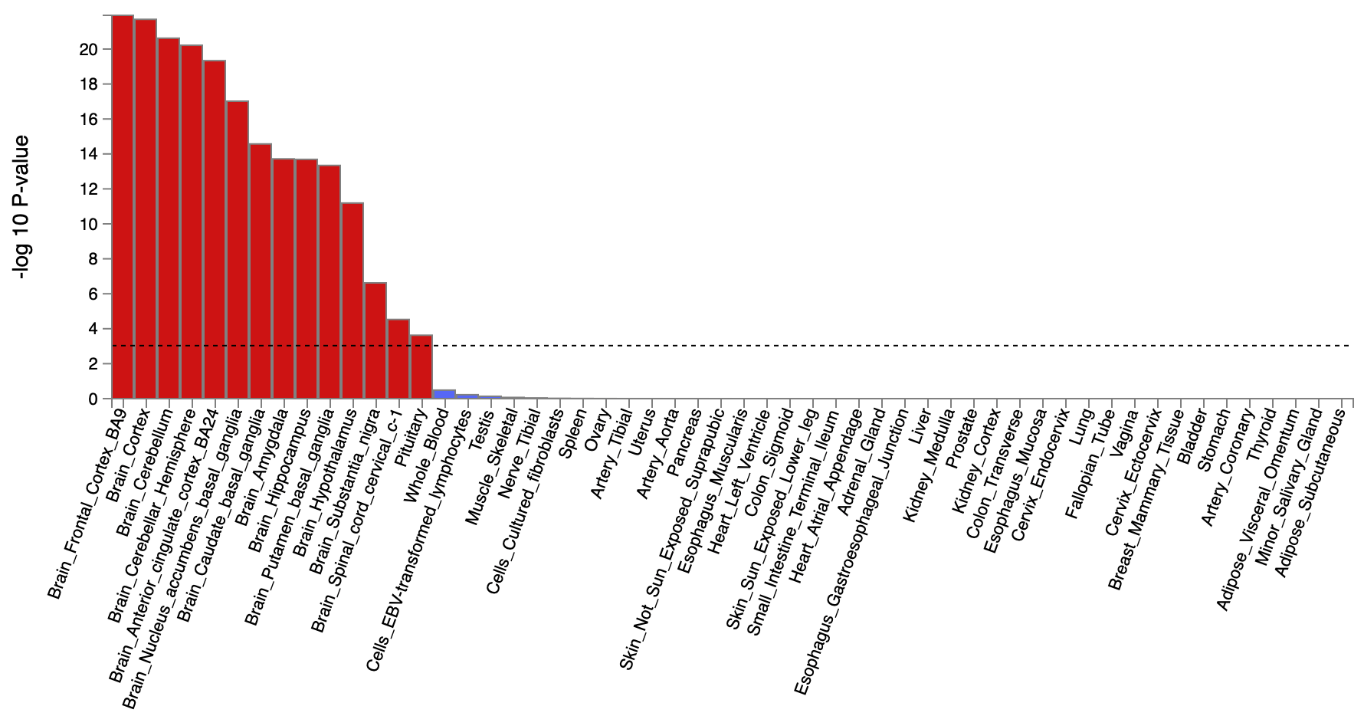


BIP corrected for p


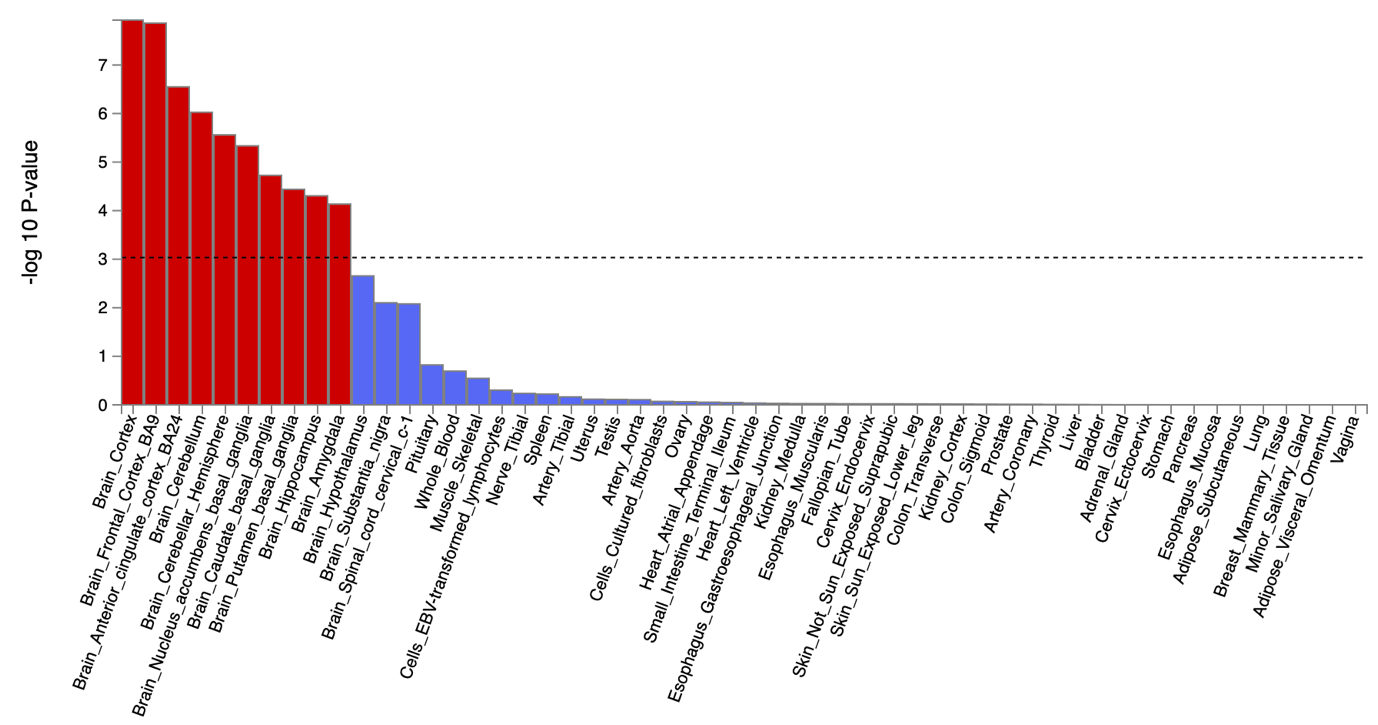


**Figure S15. The tissue type enrichment results on 53 specific tissue types by GTEx of BIP uncorrected and corrected for *p***

Results (-log10 (one-sided P-value)) from MAGMA gene-property analysis of relationships between tissue specific gene expression profiles (GTEx v.7) and BIP associations before and after correcting for *p*. The test was performed for average gene-expression per tissue type conditioning on the average expression across all categories. Dotted lines indicate significant results after Bonferroni correction. The full results are available in Supplementary Tables 24-27.


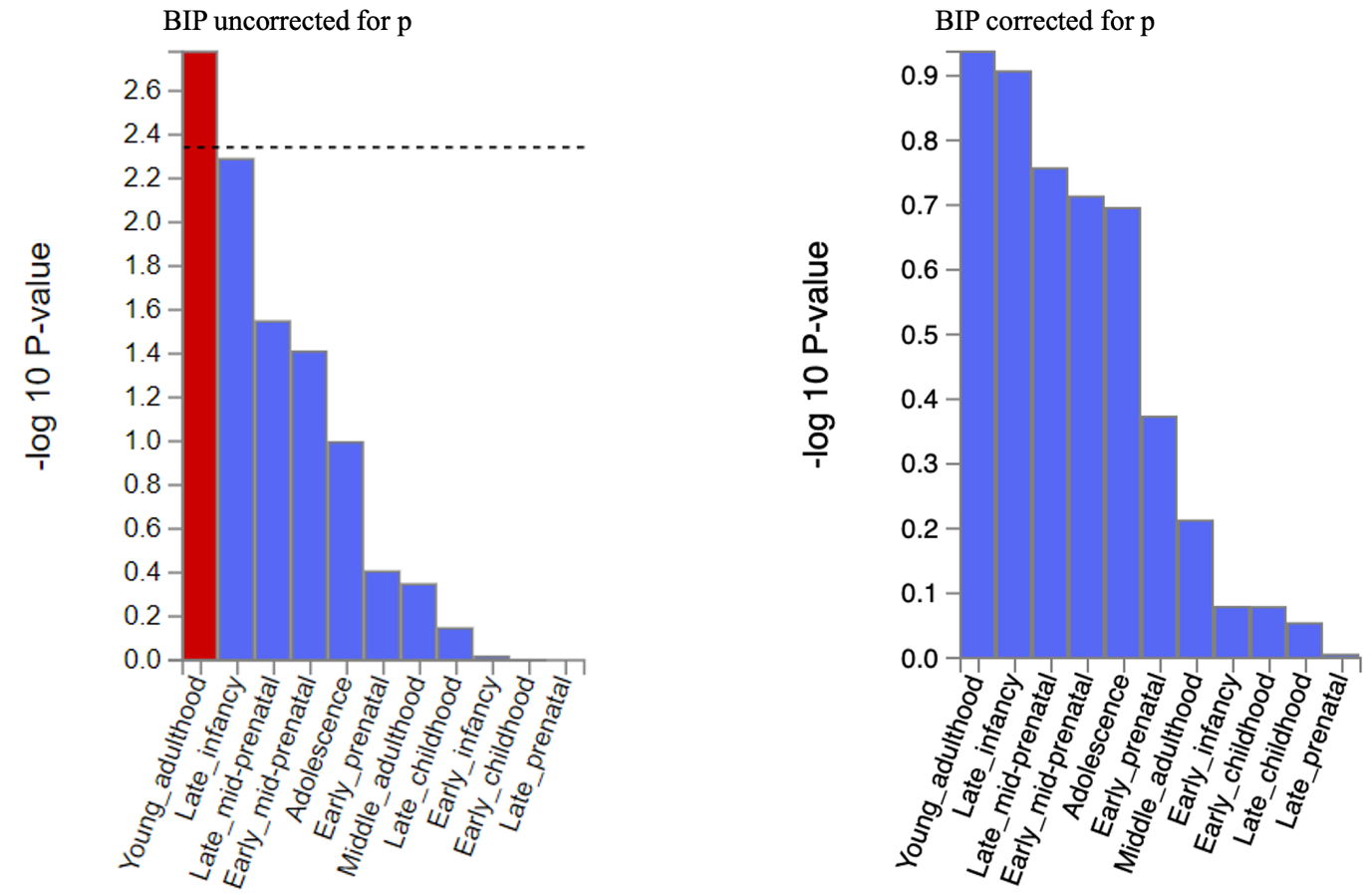


**Figure S16. The brain sample enrichment results on 11 general brain developmental stages by BrainSpan of BIP uncorrected and corrected for *p***

Results (-log10 (one-sided P-value)) from MAGMA gene-property analysis of relationships between gene expression data of developmental brain samples (BrainSpan) and BIP associations before and after correcting for *p*. The test was performed for average gene-expression per brain sample conditioning on the average expression across all developmental stages. Dotted lines indicate significant results after Bonferroni correction. The full results are available in Supplementary Tables 24-27.

SCZ uncorrected for *p*


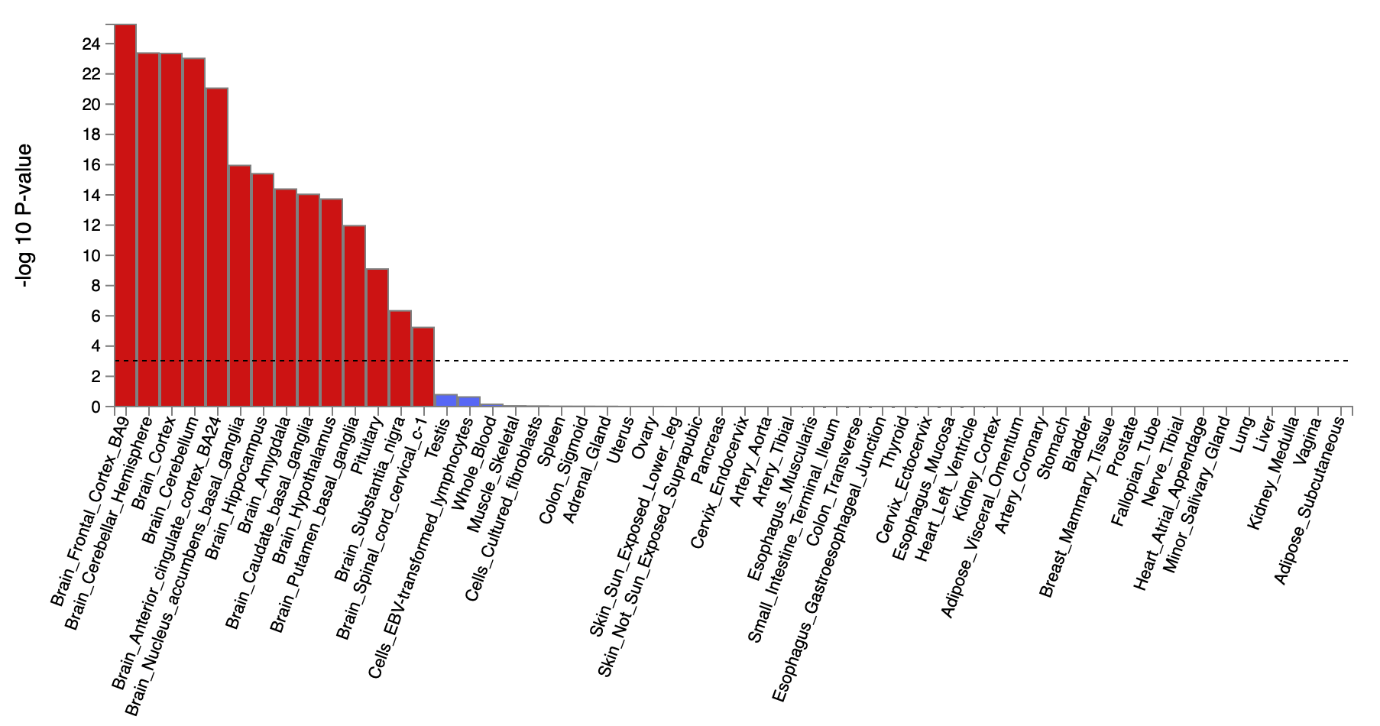


SCZ corrected for *p*


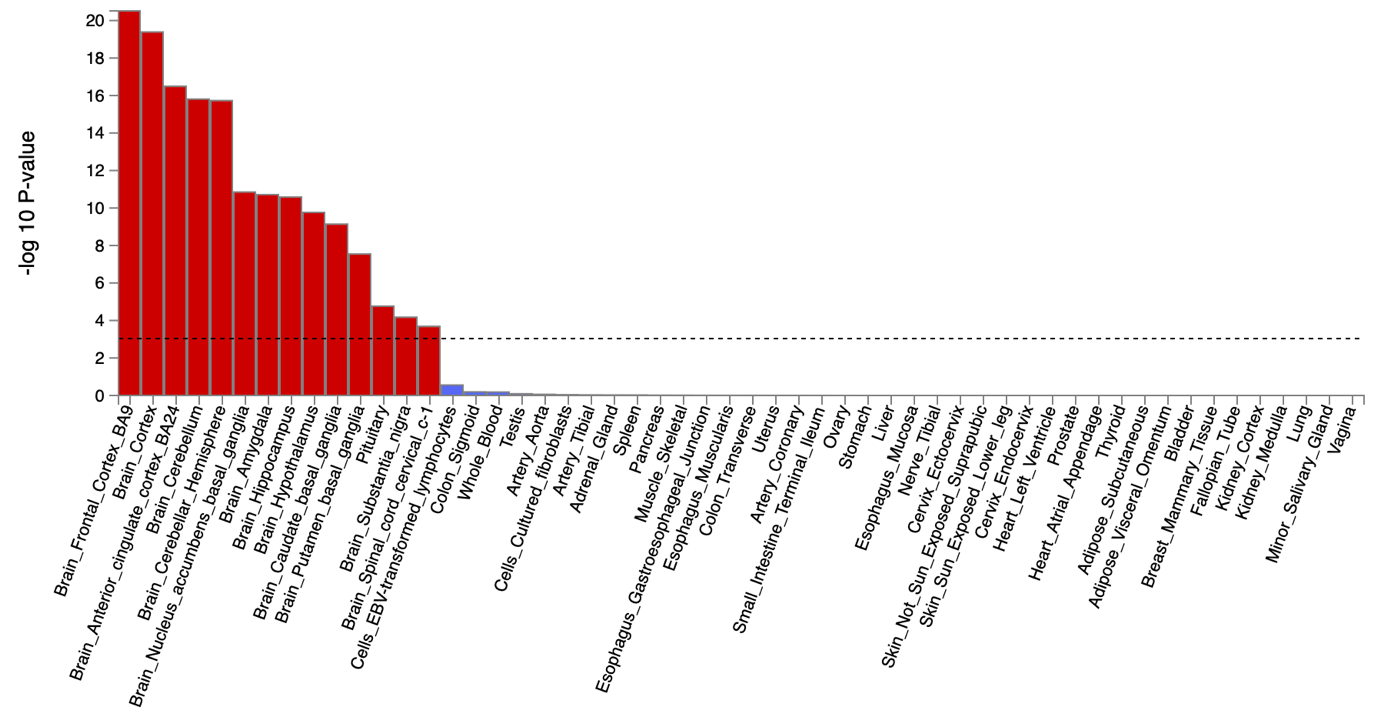


**Figure S17. The tissue type enrichment results on 53 specific tissue types by GTEx of Schizophrenia uncorrected and corrected for *p***

Results (-log10 (one-sided P-value)) from MAGMA gene-property analysis of relationships between tissue specific gene expression profiles (GTEx v.7) and schizophrenia associations before and after correcting for p. The test was performed for average gene-expression per tissue type conditioning on the average expression across all categories. Dotted lines indicate significant results after Bonferroni correction. The full results are available in Supplementary Tables 20-23.


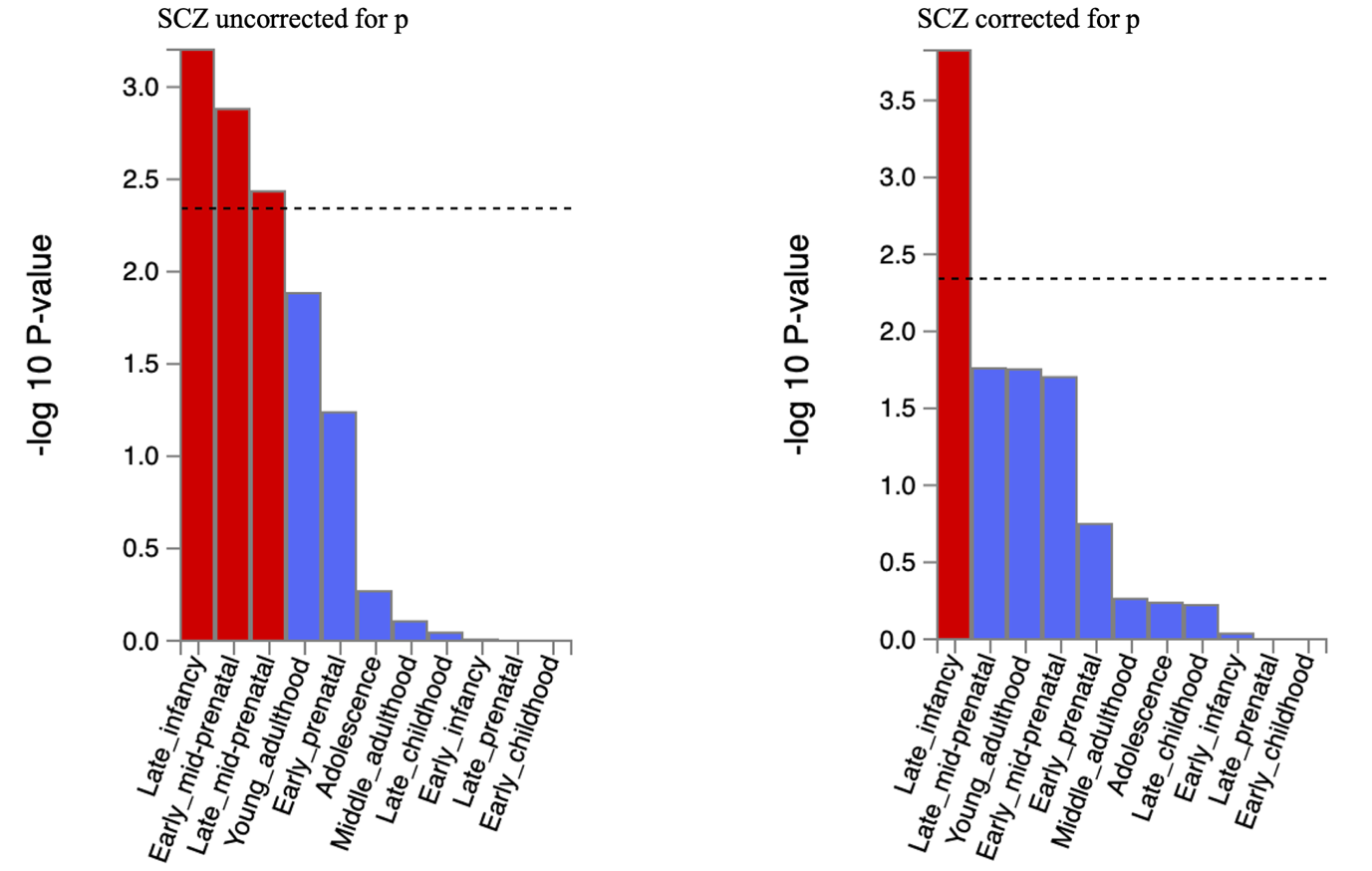


**Figure S18. The brain sample enrichment results for 11 general brain developmental stages by BrainSpan for schizophrenia uncorrected and corrected for *p***

Results (-log10 (one-sided P-value)) from MAGMA gene-property analysis of relationships between gene expression data of developmental brain samples (BrainSpan) and schizophrenia associations before and after correcting for *p*. The test was performed for average gene-expression per brain sample conditioning on the average expression across all developmental stages. Dotted lines indicate significant results after Bonferroni correction. The full results are available in Supplementary Tables 20-23.

ADHD uncorrected for p


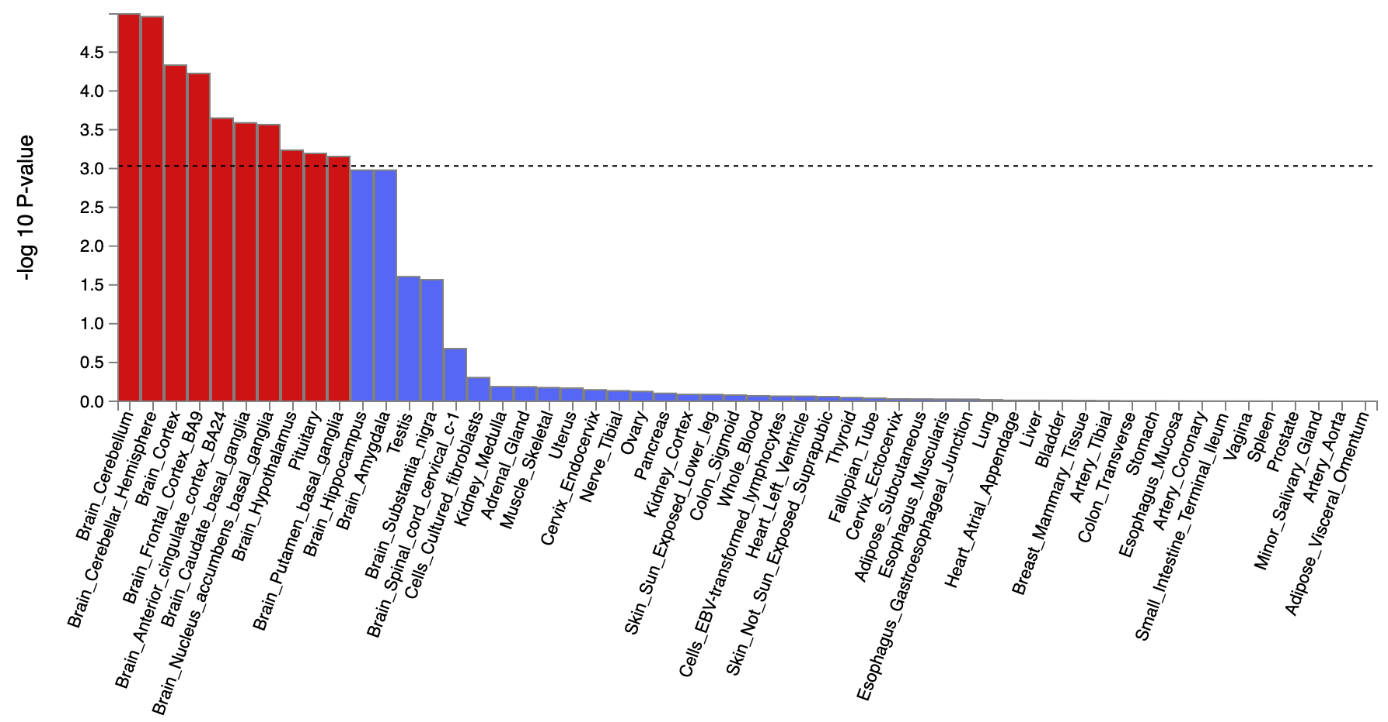


ADHD corrected for p


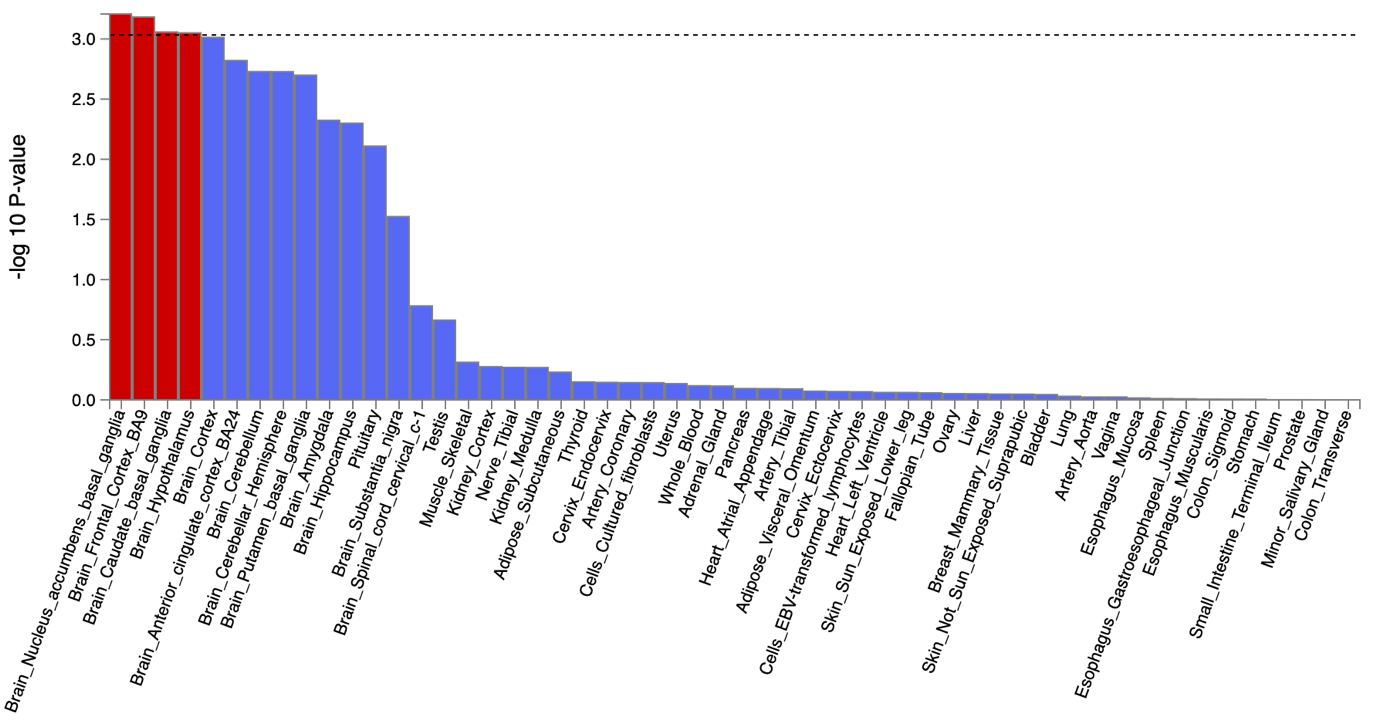


**Figure S19. The tissue type enrichment results on 53 specific tissue types by GTEx of ADHD uncorrected and corrected for *p***

Results (-log10 (one-sided P-value)) from MAGMA gene-property analysis of relationships between tissue specific gene expression profiles (GTEx v.7) and ADHD associations before and after correcting for *p*. The test was performed for average gene-expression per tissue type conditioning on the average expression across all categories. Dotted lines indicate significant results after Bonferroni correction. The full results are available in Supplementary Tables 32-35.


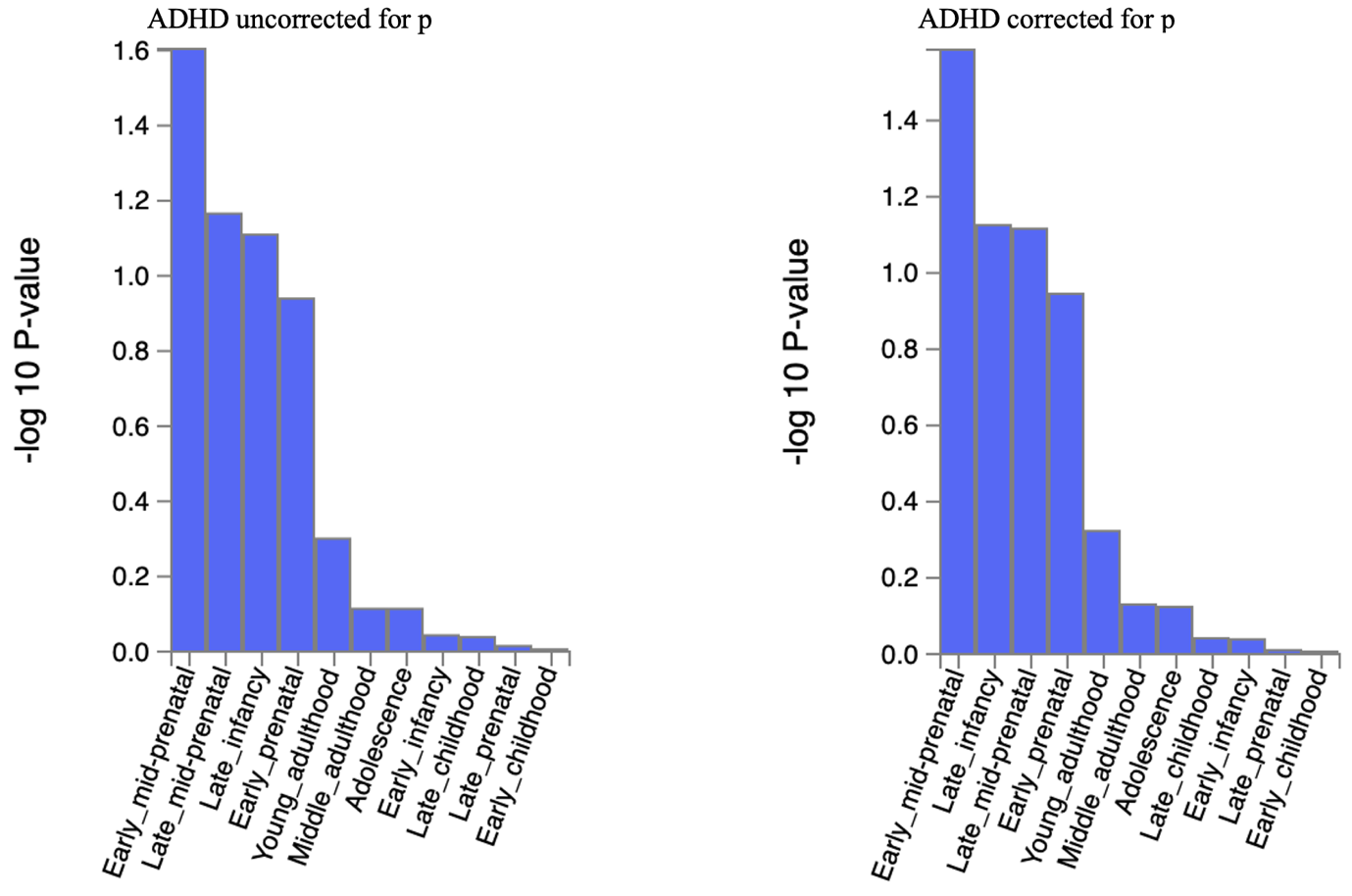


**Figure S20. The brain sample enrichment results on 11 general brain developmental stages by BrainSpan of ADHD uncorrected and corrected for *p***

Results (-log10 (one-sided P-value)) from MAGMA gene-property analysis of relationships between gene expression data of developmental brain samples (BrainSpan) and ADHD associations before and after correcting for *p*. The test was performed for average gene-expression per. brain sample conditioning on the average expression across all developmental stages. Dotted lines indicate significant results after Bonferroni correction. The full results are available in Supplementary Tables 32-35.

ASD uncorrected for p


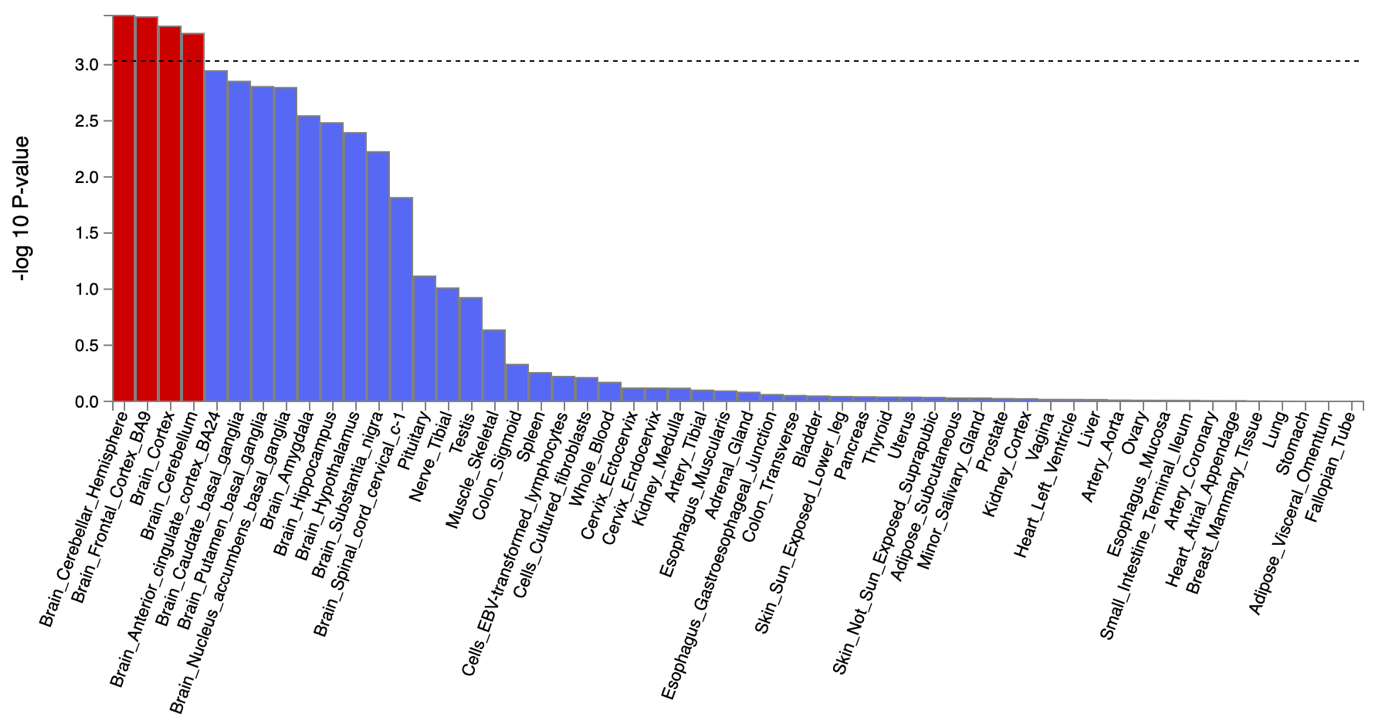


ASD corrected for p


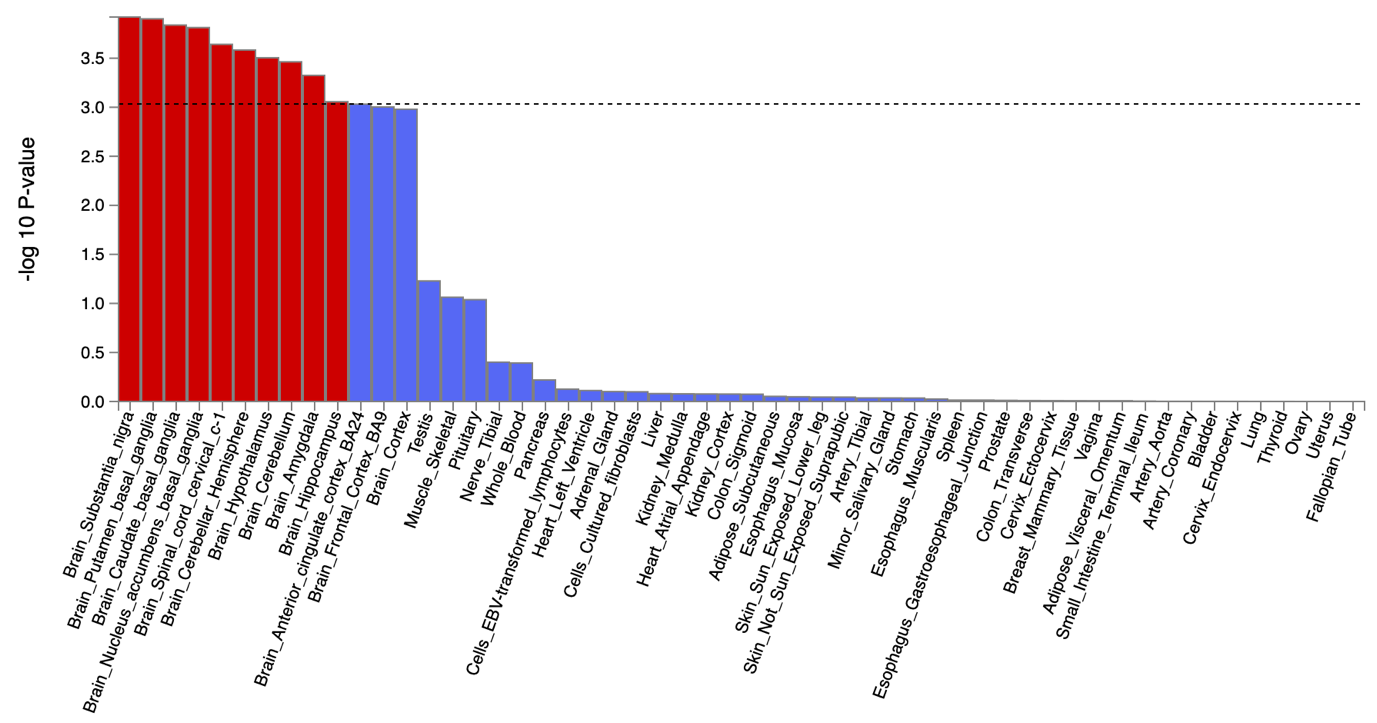


**Figure S21. The tissue type enrichment results on 53 specific tissue types by GTEx of ASD uncorrected and corrected for *p***

Results (-log10 (one-sided P-value)) from MAGMA gene-property analysis of relationships between tissue specific gene expression profiles (GTEx v.7) and ASD associations before and after correcting for *p*. The test was performed for average gene-expression per. tissue type conditioning on the average expression across all categories. The full results are available in Supplementary Tables 44-47.


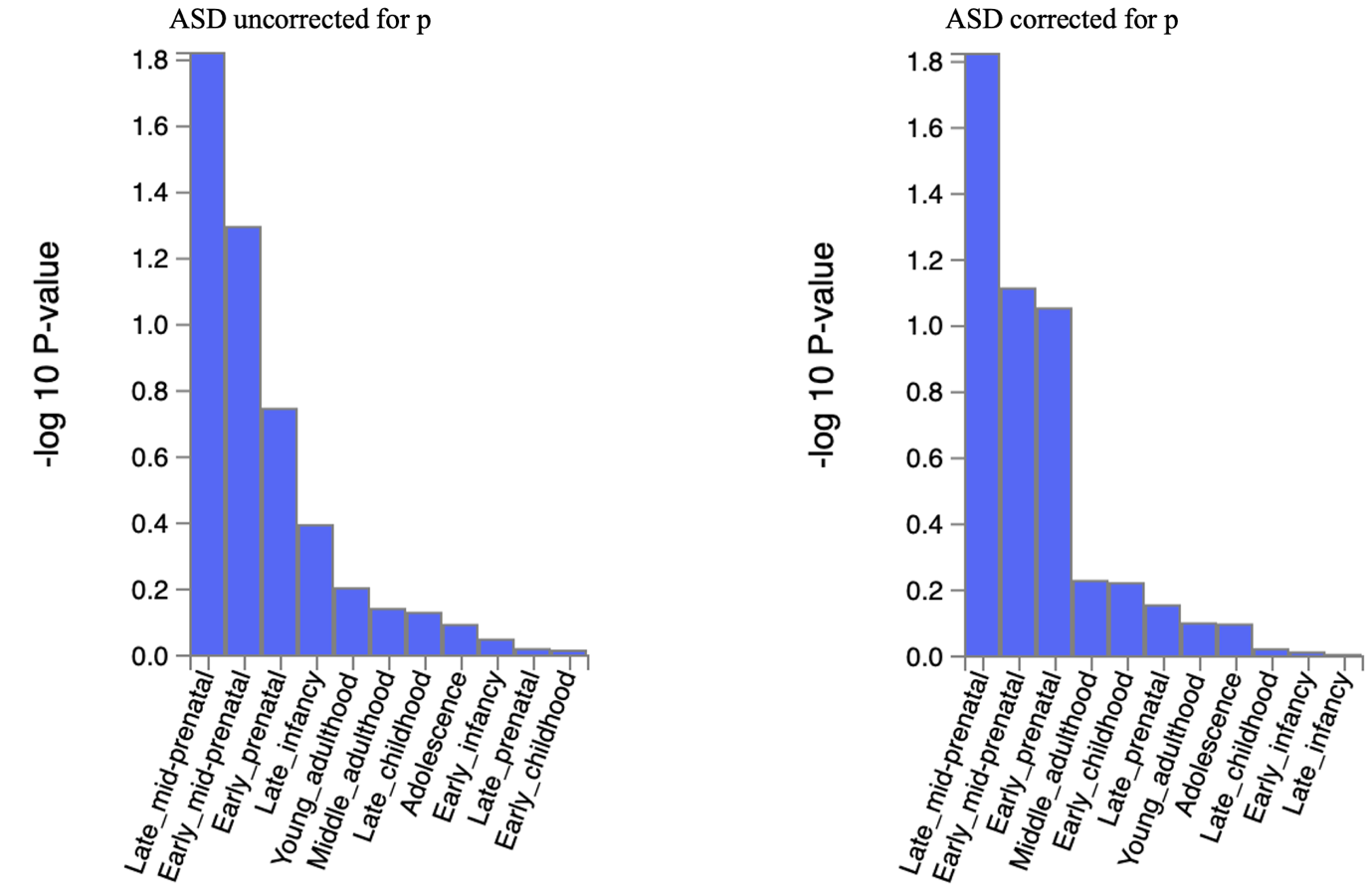


**Figure S22. The brain sample enrichment results on 11 general brain developmental stages by BrainSpan of ASD uncorrected and corrected for *p***

Results (-log10 (one-sided P-value)) from MAGMA gene-property analysis of relationships between gene expression data of developmental brain samples (BrainSpan) and ASD associations before and after correcting for *p*. The test was performed for average gene-expression per brain sample conditioning on the average expression across all developmental stages. Dotted line indicates significant results after Bonferroni correction. The full results are available in Supplementary Tables 44-47.

ALCH uncorrected for p


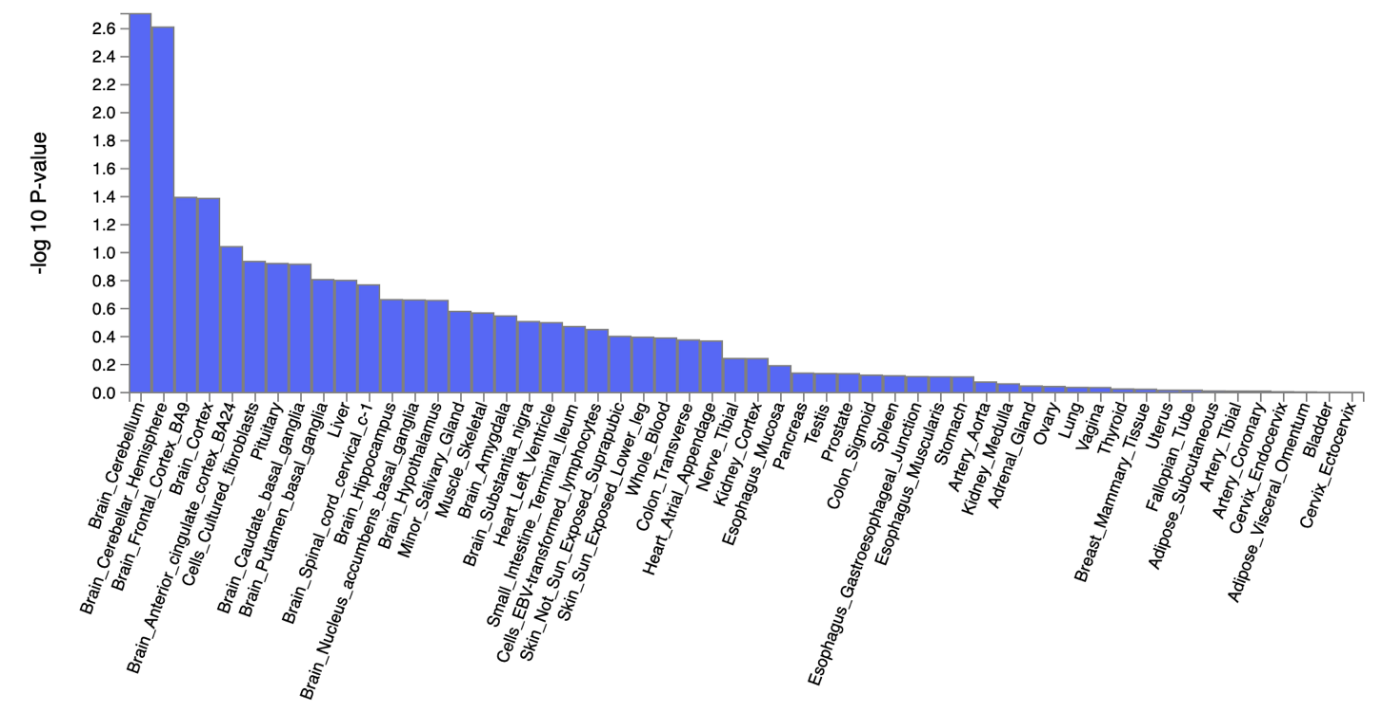


ALCH corrected for p


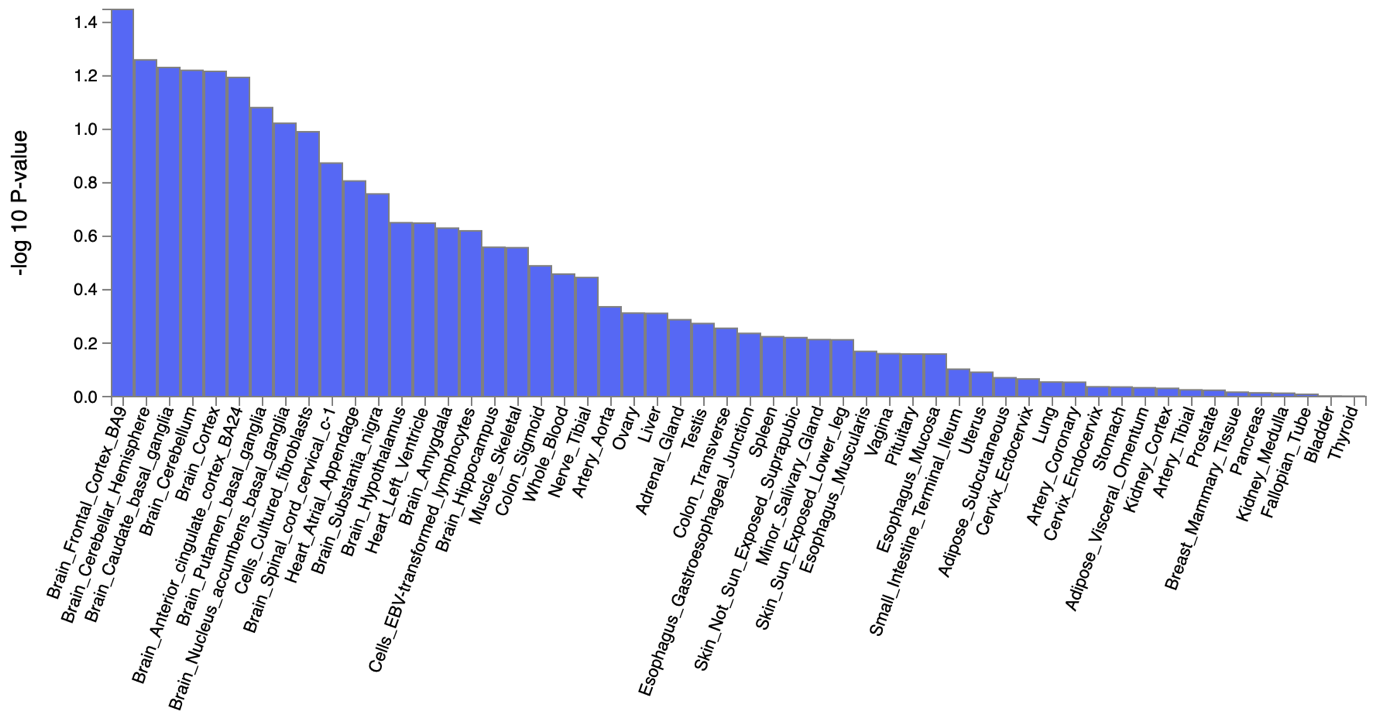


**Figure S23. The tissue type enrichment results on 53 specific tissue types by GTEx of ALCH uncorrected and corrected for *p***

Results (-log10 (one-sided P-value)) from MAGMA gene-property analysis of relationships between tissue specific gene expression profiles (GTEx v.7) and ALCH associations before and after correcting for p. The test was performed for average gene-expression per tissue type conditioning on the average expression across all categories. The full results are available in Supplementary Tables 40-43.


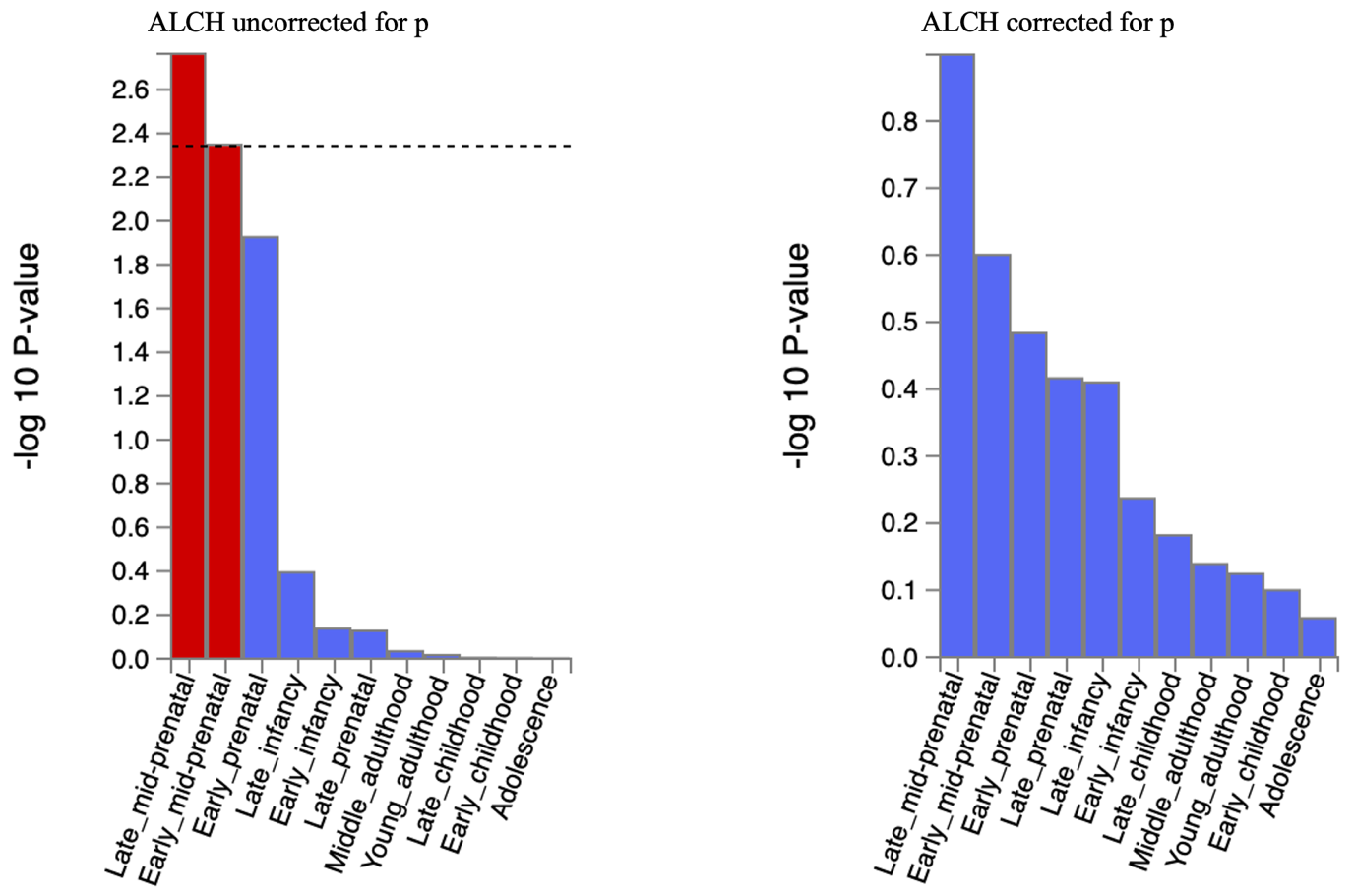


**Figure S24. The brain sample enrichment results on 11 general brain developmental stages by BrainSpan of ALCH uncorrected and corrected for *p***

Results (-log10 (one-sided P-value)) from MAGMA gene-property analysis of relationships between gene expression data of developmental brain samples (BrainSpan) and ALCH associations before and after correcting for p. The test was performed for average gene-expression per brain sample conditioning on the average expression across all developmental stages. Dotted line indicates significant results after Bonferroni correction. The full results are available in Supplementary Tables 40-43.

AN uncorrected for *p*

**
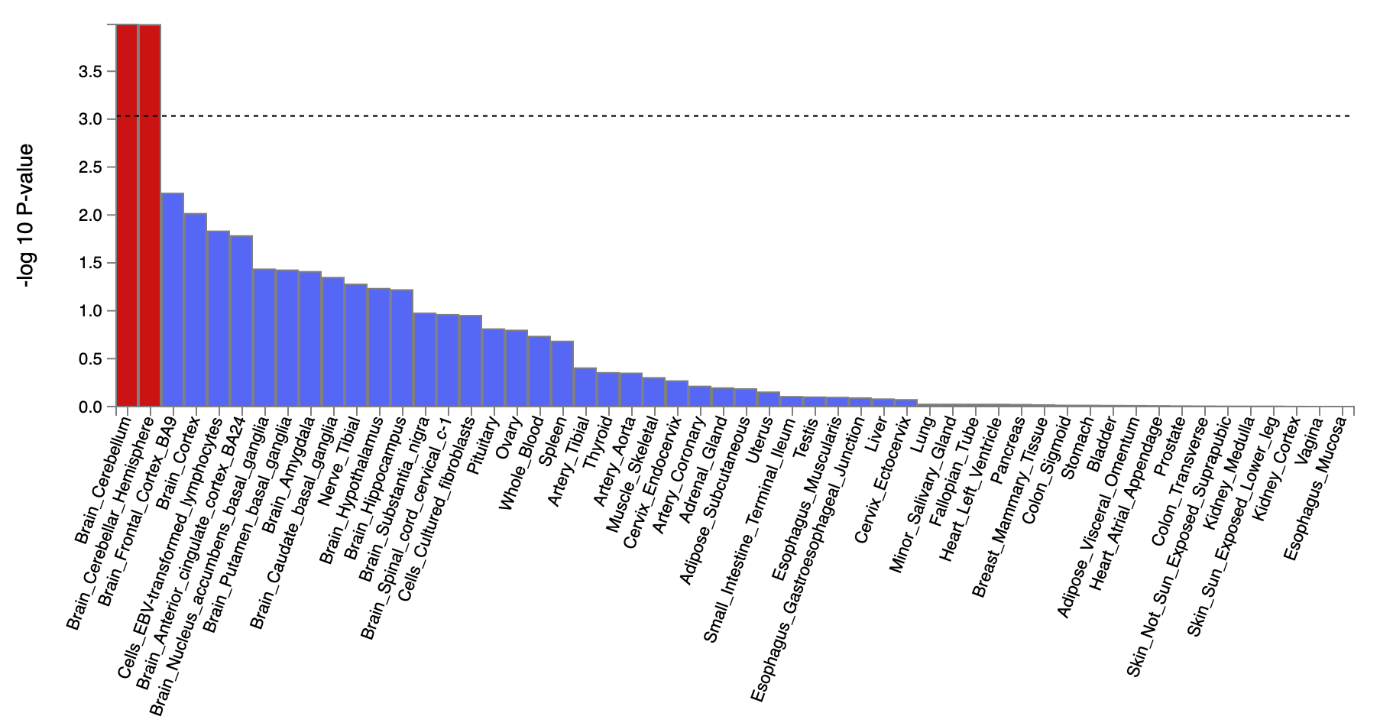
**

AN corrected for *p*


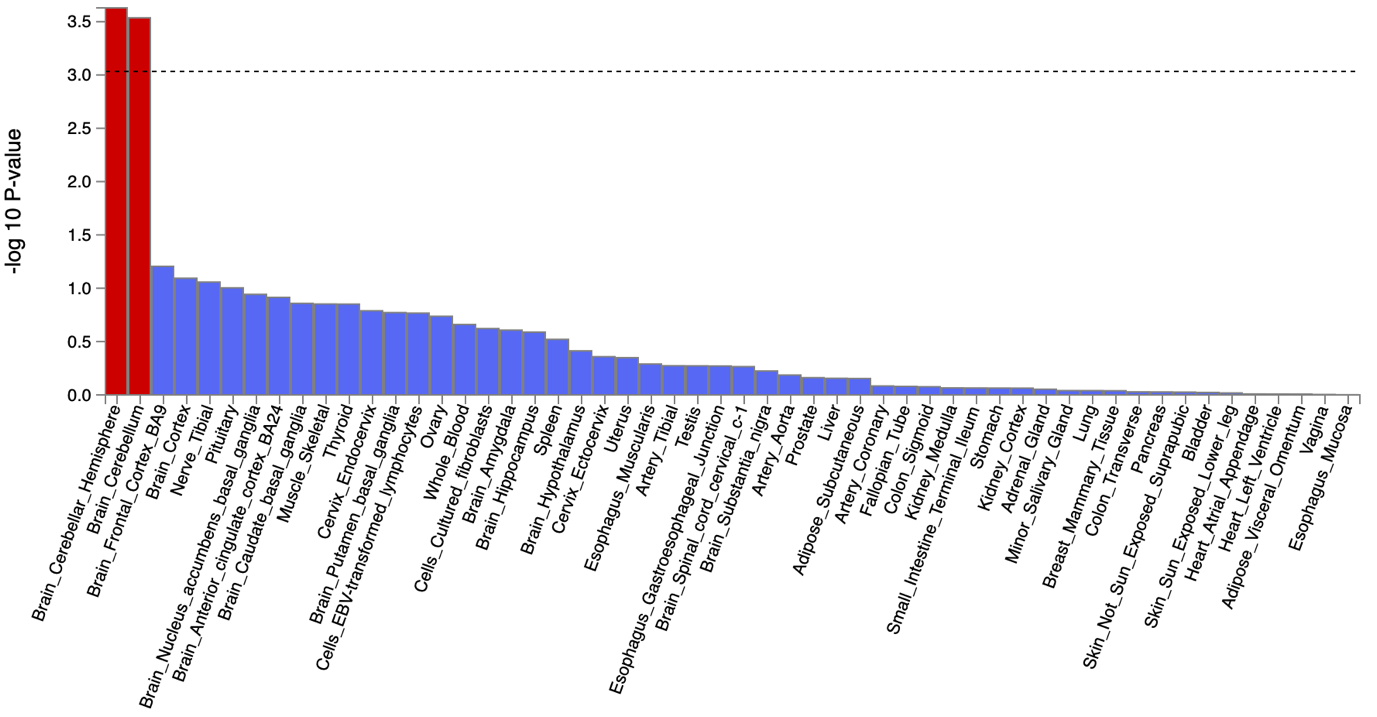


**Figure S25. The tissue type enrichment results on 53 specific tissue types by GTEx of AN uncorrected and corrected for *p***

Results (-log10 (one-sided P-value)) from MAGMA gene-property analysis of relationships between tissue specific gene expression profiles (GTEx v.7) and AN association before and after correcting for *p*. The test was performed for average gene-expression per tissue type conditioning on the average expression across all categories. Dotted lines indicate significant results after Bonferroni correction. The full results are available in Supplementary Tables 36-39.


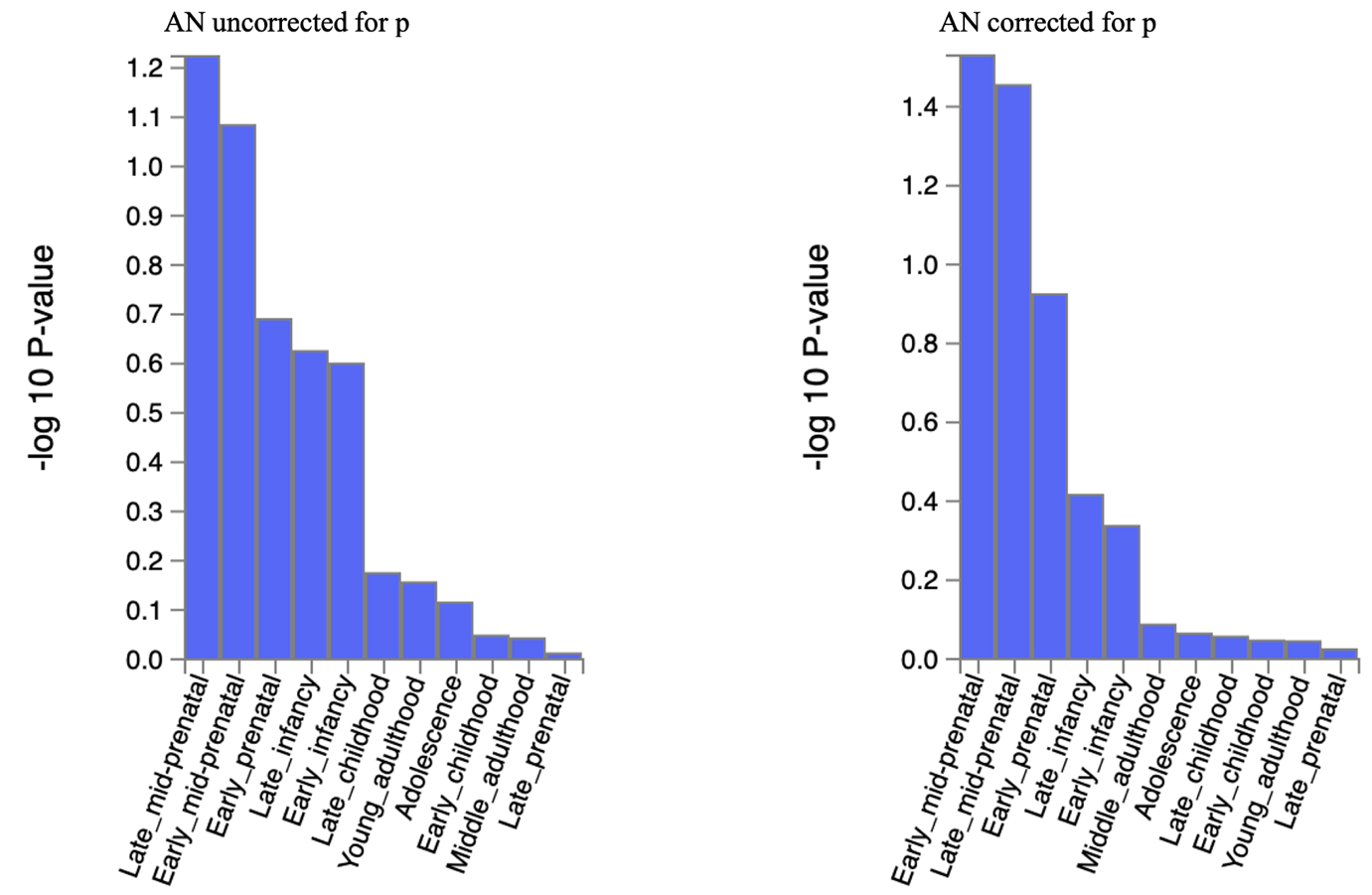


**Figure S26. The brain sample enrichment results on 11 general brain developmental stages by BrainSpan of AN uncorrected and corrected for *p***

Results (-log10 (one-sided P-value)) from MAGMA gene-property analysis of relationships between gene expression data of developmental brain samples (BrainSpan) and AN association before and after correcting for *p*. The test was performed for average gene-expression per. brain sample conditioning on the average expression across all developmental stages. The full results are available in Supplementary Tables 36-39.


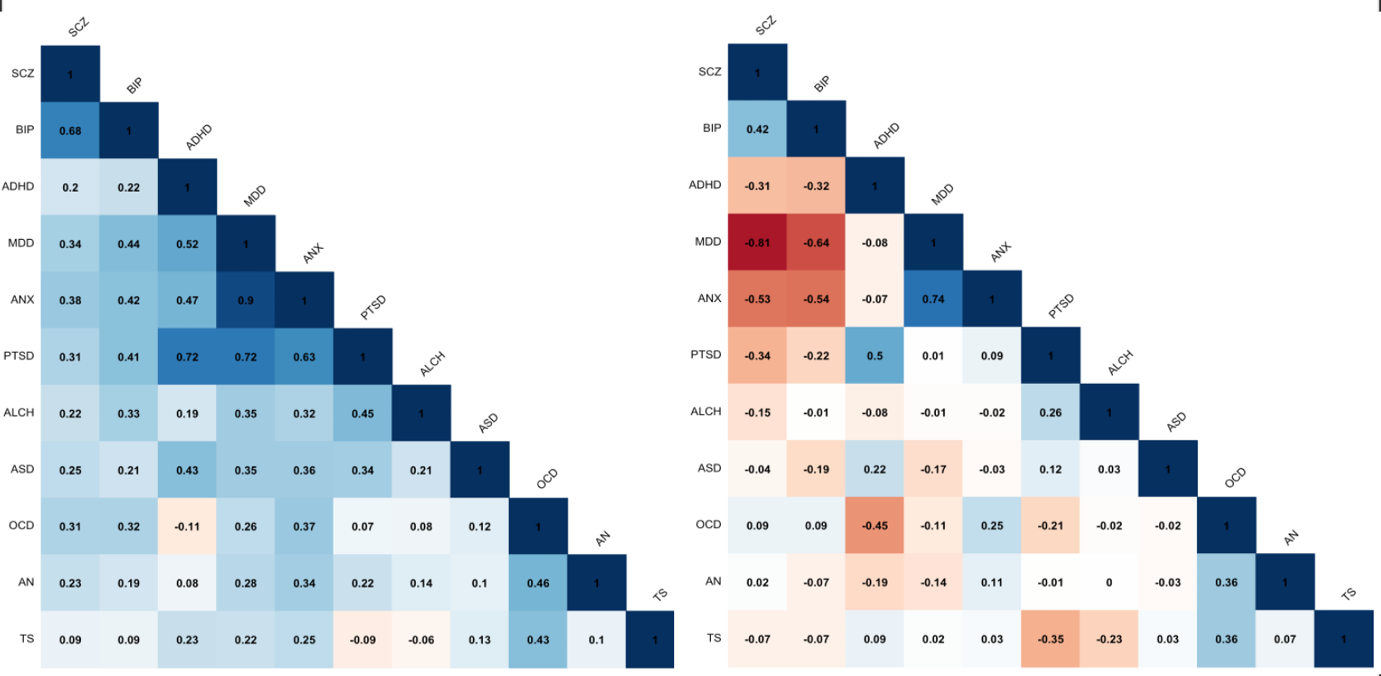


**Figure S27. Genetic correlations between psychiatric disorders before and after accounting for transdiagnostic effects obtained using the GWAS-by-subtraction modelling approach. A)** Genetic correlations between 11 major psychiatric disorders uncorrected for *p*. **b)** Genetic correlations between psychiatric disorders after removing the genetic variance each disorder shares with *p* using the two-stages approach described in Supplementary Note 3. Correlations were estimated using LDSC within Genomic SEM.

**
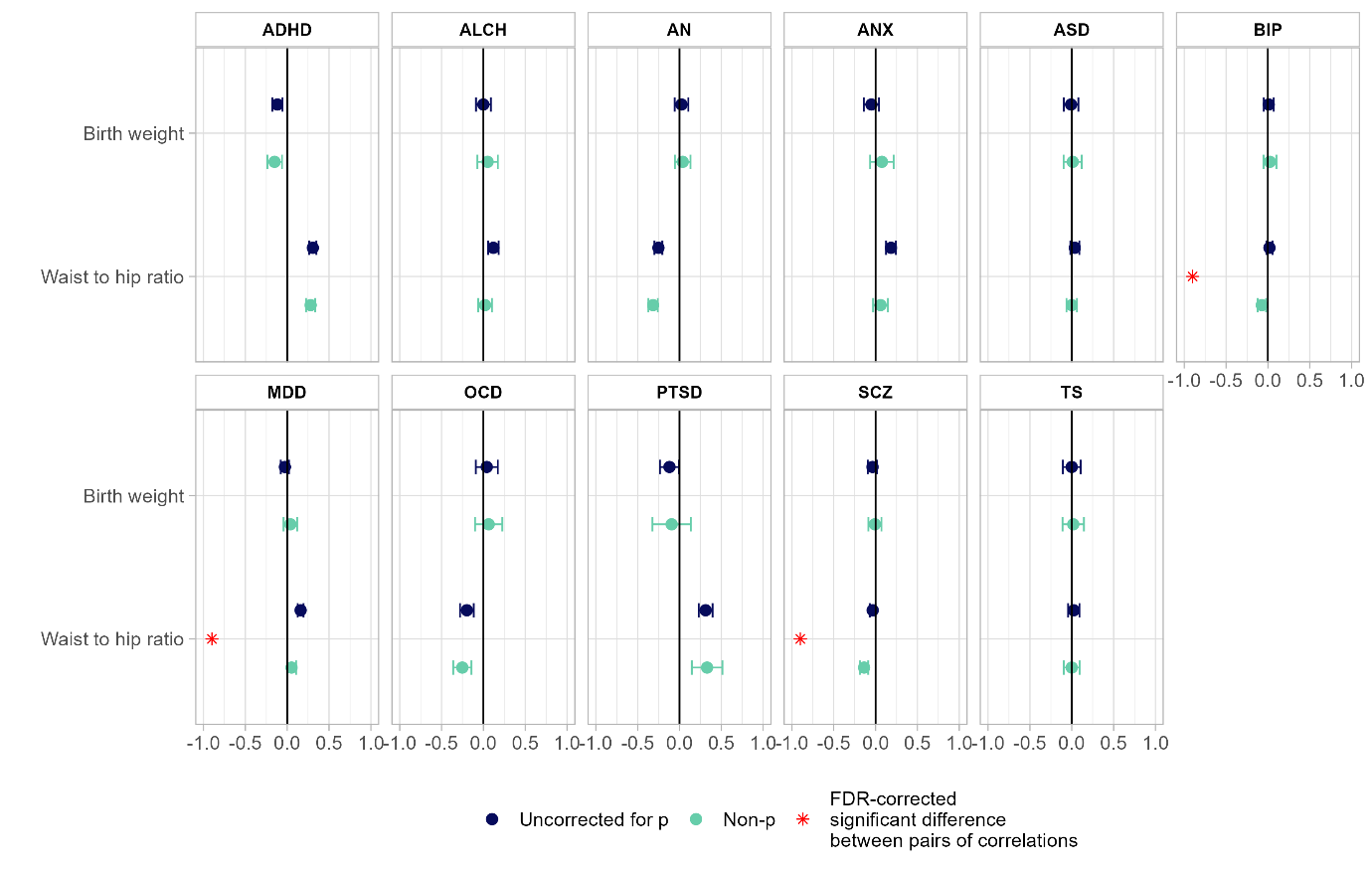
**

**Figure S28. Genetic correlations between 11 major psychiatric disorders and anthropometric traits before and after accounting for *p*.** The dots represent genetic correlations estimated using LDSC regression. Correlations with psychiatric disorders uncorrected for *p* are in blue, with psychiatric disorders corrected for *p* in green. Error bars represent 95% confidence intervals. Red asterisks indicate a statistically significant (FDR-corrected *P* < 0.05, two-tailed test) difference in the magnitude of the correlation with disorders uncorrected for p versus disorders corrected for p. Exact *P* values for all associations are reported in Supplementary Table 50.

**
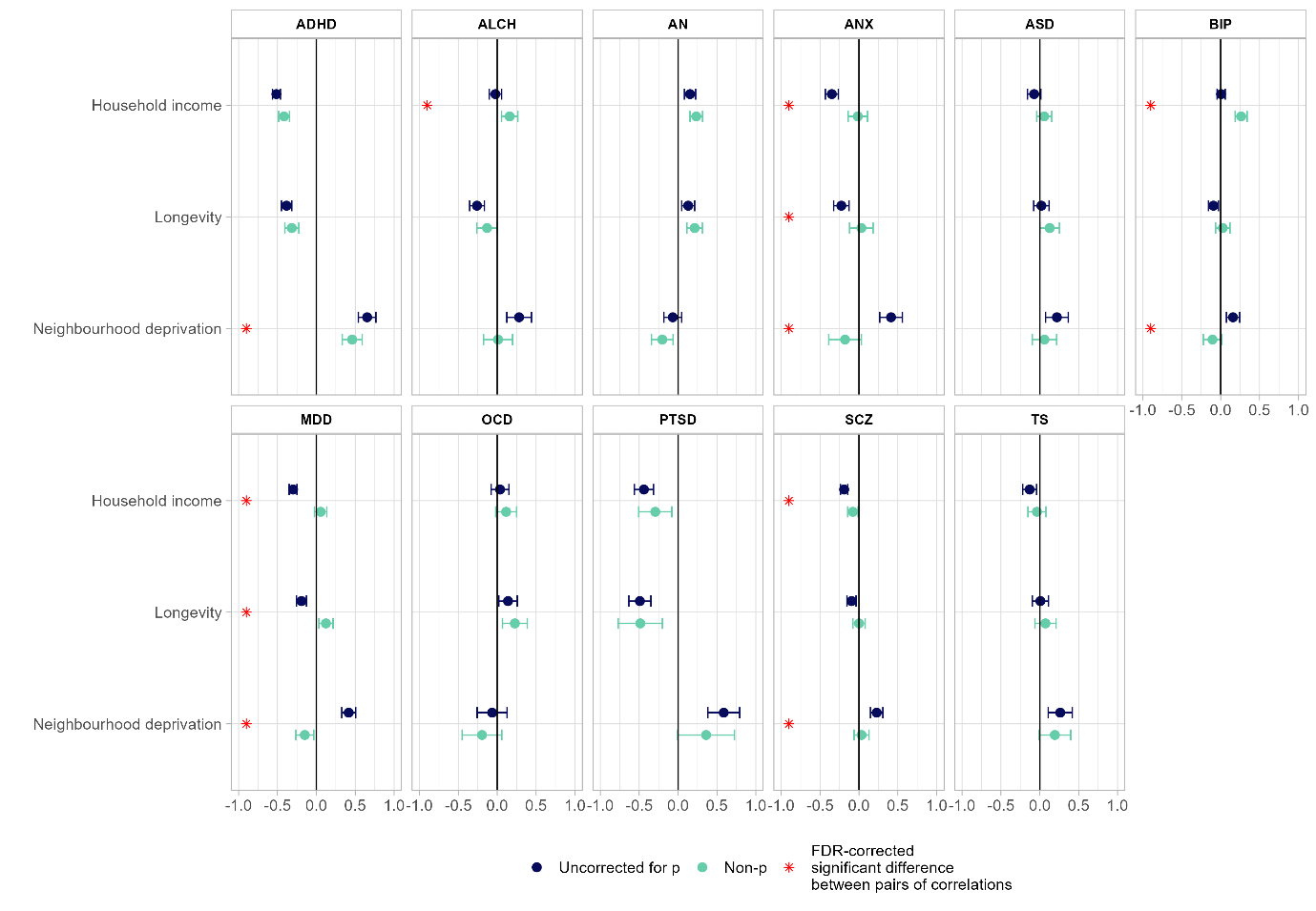
**

**Figure S29. Genetic correlations between 11 major psychiatric disorders and socio-demographic traits before and after accounting for *p*.** The dots represent genetic correlations estimated using LDSC regression. Correlations with psychiatric disorders uncorrected for *p* are in blue, with psychiatric disorders corrected for *p* in green. Error bars represent 95% confidence intervals. Red asterisks indicate statistically significant (FDR-corrected *P* < 0.05, two-tailed test) differences in the magnitude of the correlation with disorders uncorrected for p versus disorders corrected for p. Exact *P* values for all associations are reported in Supplementary Table 50.
